# Molecular Basis for Vacuolar Iron Transport by OsVIT2, a Target for Iron Biofortification in Rice

**DOI:** 10.1002/prot.26843

**Published:** 2025-05-15

**Authors:** L. B. Arend, D. S. Lima, M. G. S. Costa, F. K. Ricachenevsky, H. Verli

**Affiliations:** ^1^ Universidade Federal do Rio Grande do Sul Porto Alegre Rio Grande do Sul Brazil; ^2^ Fundação Oswaldo Cruz Rio de Janeiro Rio Grande do Sul Brazil

**Keywords:** molecular dynamics, normal mode analysis, OsVIT2, vacuolar iron transporter

## Abstract

Iron deficiency is the prevalent and most widespread nutritional shortfall for humans, affecting over 30% of the global population and leading to anemia, particularly among preschool‐aged children and pregnant women in developing countries. Simultaneously, while half of the world's population depends on rice ( *Oryza sativa* L.) as a staple food, this cereal does not provide a sufficient amount of that micronutrient to meet these people's nutritional needs: even when iron is readily available in the soil, it does not accumulate in the consumed portion of the grain, namely, the starchy endosperm, being instead retained in the aleurone layer, in the pericarp and in the embryo. In this context, the present work applies computational biology tools—such as normal mode analysis and molecular dynamics simulations—to elucidate the behavior and transport mechanism of the Vacuolar Iron Transporter 2 (OsVIT2), a central protein for iron homeostasis in rice, with the objective of laying the foundations for future OsVIT2 engineering projects that could be articulated with ongoing efforts to promote iron biofortification in rice. We shed light on the interplay between protonation state, configuration and hydration of OsVIT2's pore; on the mechanics of its opening and on the ever‐shifting hydrogen bond network contained within it. We also explore the potential contribution of the “flexible arms” to the iron‐capturing function performed by the cytoplasmic domain.

## 1 Introduction

Iron deficiency is our prevalent and most widespread nutritional shortfall, affecting over 30% of the global population and leading to anemia, particularly among preschool‐aged children and pregnant women in developing countries [1, 2]. For children, the most critical consequence of anemia is impaired psychomotor and behavioral development; for pregnant women, this condition is associated with increased perinatal risks for both mothers and newborns; as for the population, anemia impairs cognition, immunity, and physical capacity, being associated with increased morbidity [3, 4]. Simultaneously, while half of the world's population depends on rice ( *Oryza sativa* L.) as a staple food, this cereal does not provide a sufficient amount of that micronutrient to meet people's nutritional needs: even when iron is readily available in the soil, it does not accumulate in the consumed portion of the grain, namely, the starchy endosperm, being instead retained in the aleurone layer, in the pericarp, and in the embryo [5, 6, 7, 8, 9, 10].

Currently, genetic biofortification presents itself as a promising strategy to tackle this post‐green revolution “hidden hunger,” circumventing the limitations and risks of supplementation and fortification. In the context of iron biofortification in rice, the Vacuolar Iron Transporter (VIT) family—which is present not only in plants, but also in fungi and protists [10, 11, 12]—becomes an important object of study, since its members could potentially be engineered and expressed in the endosperm of a biofortified rice crop, promoting iron enrichment in the consumed portion of the grain through optimized vacuolar sequestration [13, 14, 15].

OsVIT2 has been shown to promote Fe distribution at different tissues, playing an important role in iron homeostasis in rice. More specifically, when highly expressed in response to iron excess, it seems to mediate Fe distribution from leaf sheath to leaf blade, from upper node to panicle, and from aleurone layer to endosperm and embryo [16, 17, 18]. Despite this exploration into its physiological role and the perception of its potential for biofortification, OsVIT2's structural features, behavior, and mechanism of transport remain to be elucidated. Studies centered around *Pf*VIT and *Eg*VIT1 indicate that Vacuolar Iron Transporters as a whole present an idiosyncratic fold and act as Fe^2+^/H^+^ antiporters, aided in the capturing of iron by the cytoplasmic metal binding domain. Beyond that, it was suggested that the antiport would likely implicate in the transient protonation/deprotonation of acidic residues that line the protein's pore [19, 20].

In light of these characteristics and aiming at future engineering efforts that might culminate in biofortified rice crops, we investigated OsVIT2's behavior and transport mechanism, exploring the interplay between protonation state, configuration and hydration of the pore; the mechanics of its opening and the ever‐shifting hydrogen bond network contained within it, as well as the potential contribution of the “flexible arms” to the iron‐capturing function performed by the cytoplasmic domain.

We employed computational methods in the field of molecular mechanics, such as molecular dynamics (MD) simulations and normal mode analysis (NMA). MD simulations are a well‐established tool, which has long been applied to the study of proteins and solidly developed to the point of tackling multimeric structures inserted in lipid bilayers along tens of microseconds [21, 22, 23, 24, 25], while NMA—though employed just as long in this area of study—has been gaining real traction in the last years, being applied to many enzymes, channels, and transporters and bringing to light low‐frequency motions which correspond to biologically relevant conformational changes observed by experimental analysis, as well as molecular dynamics simulations [26, 27, 28, 29, 30, 31, 32, 33, 34, 35].

## 2 Methodology

### 2.1 Protein's Structure and Systems Preparation

Firstly, OsVIT2's tridimensional structure was modeled by AlphaFold [36] via ColabFold [37], while the secondary structure of low‐confidence regions was confirmed by Jpred4 [38] and PredictProtein [39] (with Q3 scores of 82% and 76%, respectively). From this confirmed model, the p*K*_a_ of the residues was calculated by PROPKA [40].

Then, the protein + tonoplast (protein + vacuolar membrane) system was built with the aid of CHARMM‐GUI [41, 42, 43]. To determine the protonation state of ionizable residues, were taken into consideration the p*K*_a_ values calculated by PROPKA, a cytoplasmic pH of 7.5, and a vacuolar pH of 5.5 [44, 45, 46]. The protein was inserted into a phosphatidylcholine (POPC) bilayer with 160 lipids in one of the layers and 156 lipids in the other—the lipid composition of plant membranes presents a remarkable variation among tissues [46], indicating that proteins with ample distribution inside the plant function in different lipid contexts. Accordingly, since OsVIT2 is expressed in many tissues, we opted for a lipid composition as generic as possible. The TIP3 water model was used (38 866 molecules) and the system was neutralized by the addition of 10 Na^+^ and 1 Cl^−^ ions. The simulation box was built with the following dimensions: *x* ≅ 11 nm, *y* ≅ 11 nm, and *z* ≅ 15 nm.

After the minimization run by CHARMM‐GUI, the system was equilibrated using GROMACS [47, 48] and CHARMM36 force field [49, 50]. The equilibration was performed in multiple 5 ns long steps, along which the protein and lipid bilayer were progressively relaxed: in the first (NVT) and second (NPT) steps, the protein was restrained by a 5000 kJ/mol nm^2^ force constant acting upon its heavy atoms, while the membrane was restrained by a 1000 kJ/mol nm^2^ force constant acting upon its phosphorus atoms along the z axis, as per the CHARMM‐GUI protocol [41, 42, 43]; in the third step (NPT), the protein was restrained by a 4000 kJ/mol nm^2^ force constant, while the membrane was restrained by a 500 kJ/mol nm^2^ force constant; in the fourth step (NPT), 3000 and 200, and in the fifth step (NPT), 2000 and 50 kJ/mol nm^2^ force constants were applied; in the sixth step (NPT), the protein was restrained by a 1000 kJ/mol nm^2^ force constant, while the membrane was completely relaxed and, finally, in the seventh step (NPT), the restraint was limited to the protein backbone (1000 kJ/mol nm^2^). After equilibration, OsVIT2 pore and cavities were identified and characterized by MOLEonline [51].

In the sequence, three systems with alternative protonation states of the ionizable residues lining the hydrophilic pocket were generated in the following manner (Table 1): from the file resulting from the last equilibration step, which presents both Asp 39 deprotonated and both Glu 68 protonated (DE2H+), the systems were produced by the removal or addition of protons and replacement of water molecules for Na^+^ ions; they were minimized and accommodated for 1 ns (NPT) with position restraints limited to the backbone (1000 kJ/mol nm^2^). The systems in question present: both Asp 39 and one Glu 68 deprotonated (DEH+); both Asp 39 and both Glu 68 deprotonated (DE) and one Asp 39 and both Glu 68 deprotonated (DH + E).

**TABLE 1:** List of MD simulations, indicating the protonation states of the hydrophilic pocket residues, the occupation of the binding site for transition metals (MBS), and the presence of excess Fe^2+^ ions for each simulated system.

	Glu 68	Asp 39	Iron
DE2H+ (replica1)	Both protonated	Neither protonated	—
DE2H+ (replica 2)	Both protonated	Neither protonated	—
DE2H+ (replica 3)	Both protonated	Neither protonated	—
DEH+ (replica 1)	One protonated	Neither protonated	—
DEH+ (replica 2)	One protonated	Neither protonated	—
DEH+ (replica 3)	One protonated	Neither protonated	—
DE (replica 1)	Neither protonated	Neither protonated	—
DE (replica 2)	Neither protonated	Neither protonated	—
DE (replica 3)	Neither protonated	Neither protonated	—
DH+E (replica 1)	Neither protonated	One protonated	—
DH+E (replica 2)	Neither protonated	One protonated	—
DH+E (replica 3)	Neither protonated	One protonated	—
DE2H+28F	Both protonated	Neither protonated	Occupied MBS + 22 excess Fe^2+^ ions
DEH+28F	One protonated	Neither protonated	Occupied MBS + 22 excess Fe^2+^ ions
DE28F	Neither protonated	Neither protonated	Occupied MBS + 22 excess Fe^2+^ ions
AEH+	One protonated	—	—
AE2H+	Both protonated	—	—

Three systems presenting iron ions were also generated (using the FE2P model parameterized by Won [52] to reproduce the energy of solvation of Fe^2+^ and conform to the solvation shell model, via free energy perturbation; Table 1): the first, with both Asp 39 deprotonated, both Glu 68 protonated, six Fe^2+^ ions occupying the cytoplasmic transition metal binding sites and an excess of iron (22 ions) around the MBD (DE2H+28F), was produced from the file resulting from the last equilibration step (DE2H+) by the replacement of water molecules and Na^+^ ions for Fe^2+^ and Cl^−^; the system was minimized and accommodated (with position restraints applied also to the Fe^2+^ ions occupying the metal binding sites). The other two systems present both Asp 39 and either one (DEH+28F) or both (DE28F) Glu 68 deprotonated, being produced from the file resulting from the accommodation of the system DE2H+28F by the removal of protons and replacement of water molecules for Na^+^ ions; these systems were also minimized and accommodated.

Lastly, two systems with the mutant Asp39Ala presenting two alternative protonation states were generated (Table 1): one Glu 68 protonated (AEH+) or both Glu 68 protonated (AE2H+). They were produced from the files resulting from the accommodation of previously prepared systems (DEH+ and DE2H+) by the replacement of Asp 39 for alanine residues and of water molecules by Cl^−^; after adapting the topologies, the systems were minimized and accommodated. These protonation states were chosen on account of their prevalence.

### 2.2 Normal Mode Analysis

The file generated from the last equilibration step (DE2H+) was used as input for normal mode analysis (NMA), performed with R package bio3d [53, 54]. NMA consists of a computational method in the field of molecular mechanics, which is able to characterize the low‐frequency movements of a protein at a rather small computational cost, enabling the exploration of phenomena—such as the opening of a pore—that would otherwise require unfeasibly long simulations, thus circumventing the limitations imposed by MD. Normal modes can be defined as oscillatory movements along which all particles involved move at the same frequency and phase; when it comes to proteins, the set of normal modes can be understood as the decomposition of the molecule's intrinsic motions—or repertoire of movements—determined by its evolutionarily selected tridimensional structure and related to its biological function. NMA can access this repertoire through the adoption of a quadratic approximation of the free energy surface, enabling the analytic resolution of Newton's equation of movement for small motions around an energy minimum. In this sense, the protein is transformed into an elastic network and each mode behaves as a simple harmonic oscillator that presents an inverse relationship between its frequency and its amplitude/collectivity: the lower frequency modes correspond to the motion of entire domains, while the higher frequency modes correspond to the stretching of chemical bonds; between those extremes, there is an entire set of movements corresponding to the behavior of secondary structure elements, dihedral torsion, deformation of angles, and so forth [26, 27, 28, 33, 55, 56, 57, 58, 59, 60]. NMA has been applied to many enzymes, channels, and transporters, and the low‐frequency motions identified by it have been recurrently shown to correspond to biologically relevant conformational alterations observed by experimental analysis, as well as molecular dynamics simulations [26, 27, 28, 29, 30, 31, 32, 33, 34, 35].

For the calculation of OsVIT2's normal modes, we considered an elastic network composed exclusively of its alpha carbons; the first 100 modes (at the lower frequency end of the vibrational spectrum) were taken into consideration in the subsequent analysis, since the focus of this investigation was indeed the higher amplitude/collectivity motions, which might not be readily accessible through molecular dynamics simulations. They were subsequently filtered by dislocating the structure along each one of them, in both senses, up to a Root Mean Square Deviation (RMSD) value of 2 Å and selecting the modes which promote a wider distancing of the pairs of bulkier apolar residues that line up the seal (Leu 47, 51, and 60), applying a threshold of 0.5 Å. To do so, we used the R script made available in https://github.com/laisarend/OsVIT2. The average cross‐correlation of pairs of Leu 47, 51, and 60 with every residue down the protein was then calculated for each of the selected modes and a trajectory of atomic displacement was generated along each one of them, using a magnification factor of 15, to enable the visualization of the conformational changes associated with each mode.

### 2.3 Molecular Dynamics Simulations

MD also consists of a computational method in the field of molecular mechanics but, contrary to NMA, it enables the detailed simulation of a system's movements: each atom's displacement is determined—from a step to the next—through the resolution of the equation associated with Newton's Second Law of Motion. The resulting force (*F*), which acts upon each atom in each step, corresponds to the sum of every inter‐ or intramolecular force to which the atom is submitted because of its participation in bonds, angles, and dihedrals (bonded terms) and Van der Waals or electrostatic interactions (nonbonded terms). The set of bonded and non‐bonded terms, along with their associated parameters (that are calibrated through experimental data or quantum mechanical calculations), are called a “force field,” which describes the relationship between a given set of atomic coordinates and its potential energy [61].

Here, four systems with alternative protonation states of the ionizable residues lining the hydrophilic pocket were simulated (triplicates); in the sequence, three systems presenting iron ions were simulated; lastly, two systems with the mutant Asp39Ala presenting two alternative protonation states were simulated (Table 1).

The molecular dynamics simulations were run with GROMACS and CHARMM36 force field; they lasted 1 μs each, with an integration time of 2 fs, the employed integrator being a leap‐frog algorithm. Every bond was treated as a holonomic constraint and the LINCS method was utilized to solve them (lincs_iter = 1 and lincs‐order = 4). The PME method was employed to deal with the nonbonded interactions, while the Verlet method was used as a cut‐off scheme (nstlist = 5; rlist = 1.2; rcoulomb = 1.2; rvdw = 1.2; pme_order = 4; fourierspacing = 0.16; Dispcorr = EnerPress). The temperature of the system was kept constant by the Nosé‐Hoover thermostat, two coupling groups being used for this end, one of them containing protein and lipid bilayer and the other containing water and ions; the reference temperature and time constant utilized were 300 K and 0.5 ps, respectively. As for the pressure of the system, it was kept constant by the Parrinello‐Rahman barostat, applying a semi‐isotropic coupling, so that the z axis is independent from the *x* and *y* axes (tau_p = 2.0; ref_p = 1.0; compressibility = 4.5e−5). The comm‐mode = Linear and nstcomm = 100 options were utilized to remove the translational center of mass motion of two groups, one containing protein and lipid bilayer and the other containing water and ions, avoiding their sliding in relation to each other. Periodic boundary conditions were applied in every direction.

Finally, the produced trajectories were analyzed using resources of GROMACS itself, as well as R packages bio3d and mclust [62]; the systems were compared with respect to the conformation and hydration of the pore, the behavior of the cytoplasmic portion of TM1, and also protein dynamics as a whole. When a distinction was observed between alternative protonation states (as was the case for the conformation and hydration of the pore), triplicates were taken into consideration to increase the robustness of our discussion. The potential energy was calculated along each simulation to evidence the stability of the simulated systems (Figure S9).

### 2.4 Sequence Alignment

A blast search was performed, inputting OsVIT2's sequence as query against NCBI's non‐redundant protein database. From the aligned sequences, the 5000 which presented higher identity were downloaded and used as input for Multiple Sequence Alignment in MEGA [63] employing ClustalW [64] algorithm. The logos were generated with WebLogo3 [65].

## 3 Results and Discussion

### 3.1 OsVIT2's Tridimensional Structure

**FIGURE 1:**
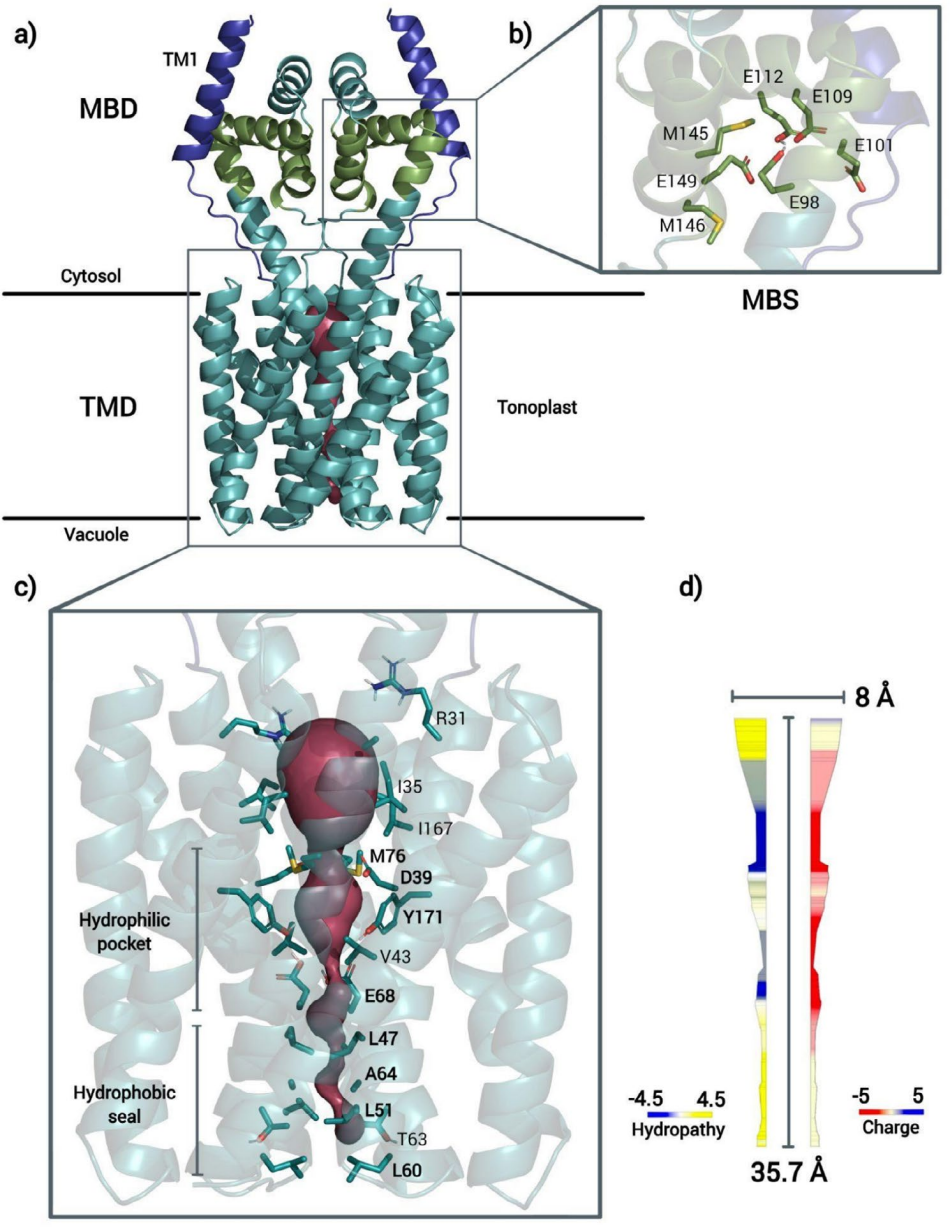
(a) OsVIT2 model, highlighting the cytoplasmic portion of TM1 (purple), the MBS (green) and the pore (pink). (b) Zoom into the MBS, showing the residues, in H1 and H3, that constitute it. (c) Zoom into the transmembrane region, showing the pore and the residues that constitute it; the pore is slightly strangulated by Asp 39 and Tyr 171 at the entrance of the hydrophilic pocket, and by the bulkier apolar residues that line up the hydrophobic seal (Leu 47, 51, and 60), being completely cut off from the vacuolar side by Leu 60. (d) Schematic representation of OsVIT2's pore, colored by hydropathy (on the left) or charge (on the right) and indicating its dimensions.

According to Kato et al. [20], the crystal structure of *Eg*VIT1 presents a novel fold, which differs from those of any transporter structures reported and is likely to be conserved among the members of its family. Indeed, the model generated for OsVIT2 presents the same idiosyncratic fold, as well as the same pore architecture as *Eg*VIT1: the protein is a homodimer that assembles into a cytoplasmic metal binding domain (MBD) and a transmembrane domain (TMD). Each protomer comprises 5 transmembrane segments (TM1‐5) organized around TM1. TM1, TM2, and TM3 extend beyond the tonoplast on the cytoplasmic side, TM2 being connected to TM3 by 3 short cytoplasmic helices (H1‐3). The cytoplasmic extremity of TM2, together with H1 and H3, creates a binding site for transition metals (one for each protomer; MBS), while in the dimer interface, both protomers create a cavity open to the cytoplasm, which extends until the middle of the transmembrane region, where we find a hydrophilic pocket composed of a pair of aspartic acid residues (Asp 39, TM1) and a pair of methionine residues (Met 76, TM2) at the entrance, followed by a pair of tyrosine residues (Tyr 171, TM3) and, finally, a pair of glutamic acids (Glu 68, TM2) at the bottom. Next to the hydrophilic pocket, the pore is obstructed by a hydrophobic seal made up of a series of apolar residues, which extends until the vacuole (Figure 1).

The hydrophilic pocket and hydrophobic seal residues are overall highly conserved, showing no variability whatsoever for Asp 39 and Met 76 and very low variability for Leu 47 and 51, Glu 68, and Tyr 171. While the outermost hydrophobic seal residue, Leu 60, presents a higher variability, it is still invariably apolar, with leucine, isoleucine, and valine being apparently interchangeable (Figure S1).

When obtaining EgVIT1's crystal structure, Kato et al. worked with a truncated version of the protein EgVIT1_23‐248_, which lacked the cytoplasmic portion of TM1, an admittedly flexible segment [20]. In the model generated for OsVIT2, this was in fact the only region with low confidence, and its secondary structure has been confirmed as inherently disorganized by Jpred4 [38] and PredictProtein [39] (Figures S2 and S3).

### 3.2 Characterization of Pore‐Opening Motions by NMA

**FIGURE 2:**
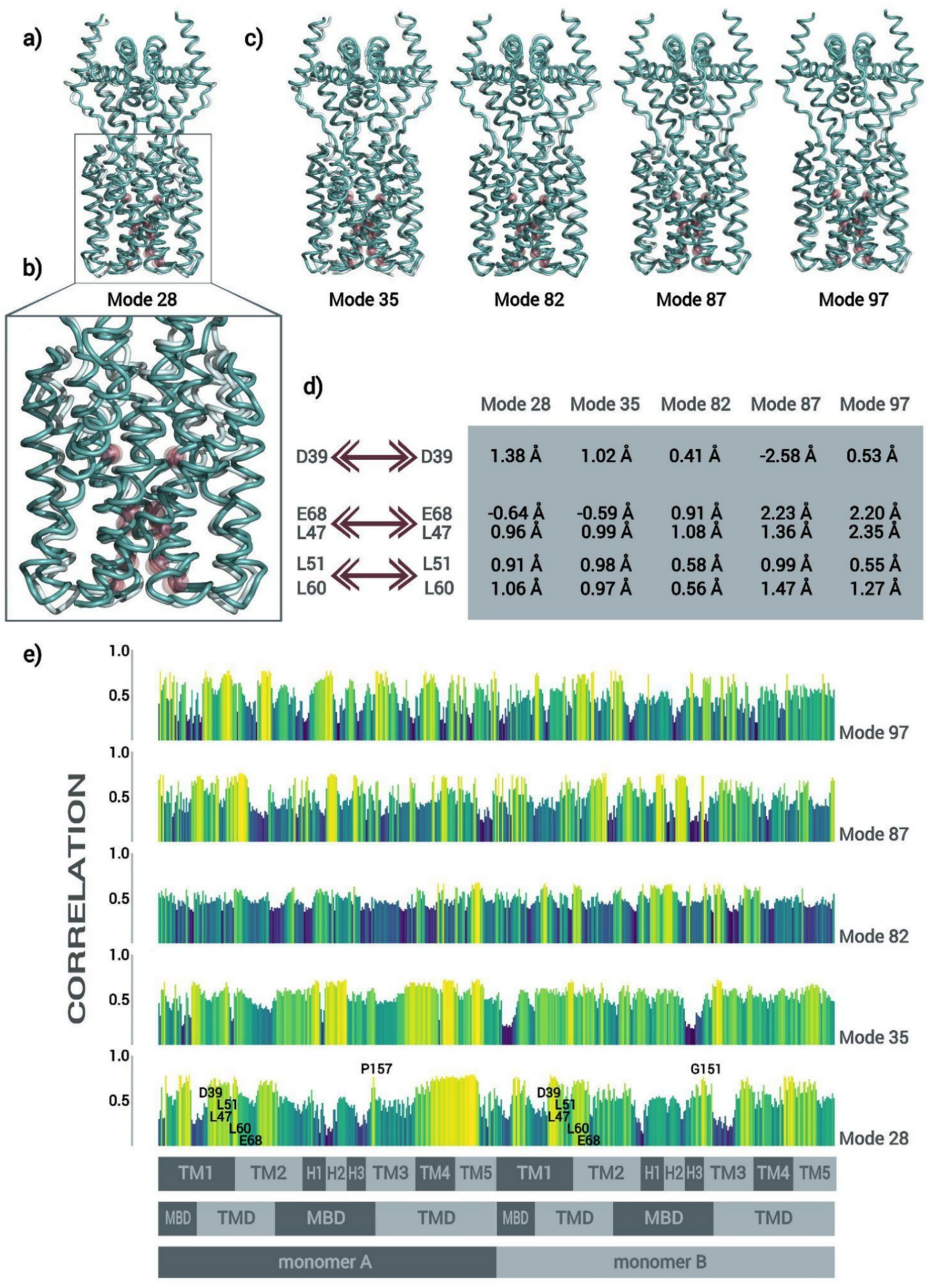
(a) Superimposition of the most extreme configurations assumed by OsVIT2 along the trajectory of atomic displacement generated for normal mode 28; spheres indicate Leu 47, 51, and 60, Asp 39 and Glu 68. (b) Zoom into the transmembrane region, evidencing the subtlety of the conformational changes observed in the pore. (c) Superimposition of the most extreme configurations assumed by OsVIT2 along the trajectory of atomic displacement generated for the subsequent normal modes. (d) Table showing the distancing between the pairs of Leu 47, 51, and 60, Asp 39 and Glu 68, when the structure is dislocated up to a RMSD value of 2 Å along each of the 5 modes. (e) Average correlation of pairs of Leu 47, 51, and 60 with every residue down the protein for each mode.

The opening of OsVIT2's pore, obstructed by the hydrophobic seal, was first explored via NMA. As previously detailed, a filtering protocol was applied to select the modes which promote a wider distancing of the pairs of bulkier apolar residues that line up the seal (Leu 47, 51, and 60) and this resulted in only five modes, which promote the concomitant distancing of the pairs of Leu 47, 51, and 60 and the pairs of Asp 39 and Glu 68 (Figure 2), suggesting a coordinated mechanism in which conformational changes in the hydrophilic pocket might propagate to the hydrophobic seal. Beyond that, as can be seen in Figures 2 and Figure S4, the subtle conformational changes of the pore are diffusely integrated to the rest of the protein in highly collective motions which do not, however, bring about any major relative dislocations between domains, segments or sites (and which allude to the expanding and contracting of lungs).

The subtlety of the conformational changes observed in the pore is consistent with the fact that the opening of the hydrophobic seal must enable the passage of Fe^2+^ (which presents a diameter of ≅1.54 Å [66, 67]) and the counter flux of H^+^, likely, through an ephemeral water bridge (each H_2_O molecule presenting an approximate diameter of 2.57 Å [68, 69]), while, at the same time, preventing proton leaks. It must be pointed out that, even though the actual magnitude of the conformational changes associated with the opening of the pore is not accessible through this type of analysis (since the different modes may act synergistically or antagonistically to each other and the movements described by each of them individually may not be energetically viable beyond a certain extent or in one of the senses), NMA did identify a type of conformational change with little wiggle room for a much wider opening of the pore: the distancing of the above‐mentioned pairs of residues is a consequence of a spiral movement of TM1 and TM2 around the pore's axis, which would not give rise to wider openings even when magnified. In this context, an eventual leeway to the antiport could be promoted by the Leu 47, 51, and 60 sidechains, which are much more flexible than the backbone and probably enable a wider opening of the hydrophobic seal.

As for the diffuseness of the integration between the conformational changes observed in the pore and the rest of the protein, we do observe some stronger correlation between the pairs of bulkier apolar residues that line up the seal and some transmembrane regions of the protein and some specific residues—Pro 157 and Gly 157—in the loops that connect H3 to the transmembrane portion of TM3 along normal mode 28; normal mode 35 presents a not so different correlation profile, with the exception of a higher correlation between the pairs of bulkier apolar residues that line up the seal and the short cytoplasmic helices (which comprise the MBS). Other than that, the 3 normal modes with higher frequency (82, 87 and 97) present comparatively leveled and regular profiles (Figure 2).

### 3.3 Conformational Changes, Hydration and Protonation States of the Pore

**FIGURE 3:**
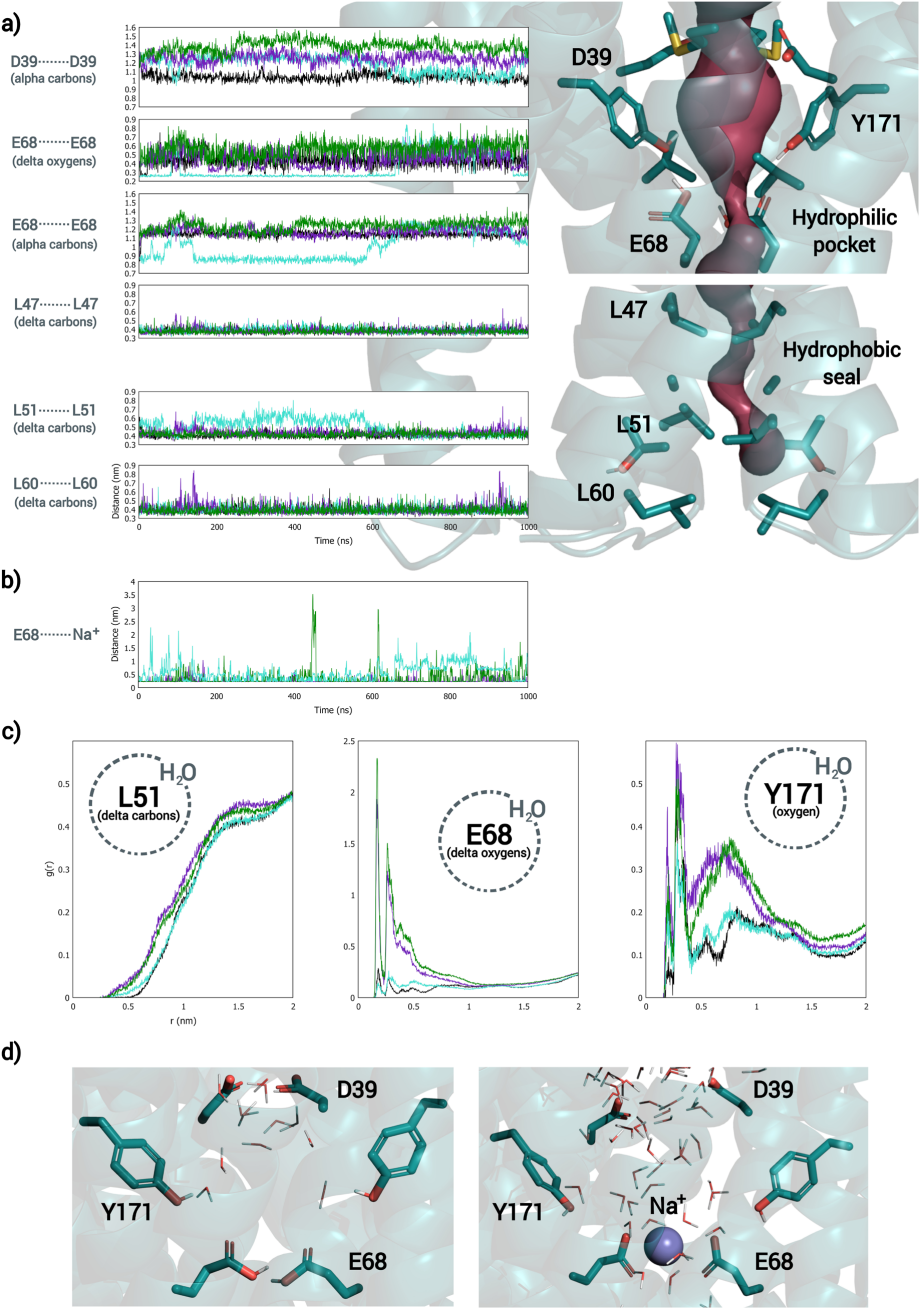
(a) Variation of the distance between pairs of ionizable residues that constitute the hydrophilic pocket and pairs of bulkier apolar residues that line the hydrophobic seal along 1 μs long MD simulations; each line color corresponds to a protonation state of the ionizable residues that constitute the hydrophilic pocket (DE2H+ in black; DEH+ in cyan; DE in purple and DH+E in green). (b) Variation of the distance between Glu 68 and the nearest Na^+^ ion along the trajectories. (c) RDF plots for the same trajectories, showing the hydration layers of Glu 68 and Tyr 171 but consistent lack of hydration of Leu 51. Replica 1. (d) Frames from MD trajectories displaying the presence of water molecules inside the hydrophilic pocket to illustrate the difference between two protonation states (DE2H+ on the left and DE on the right).

**FIGURE 4:**
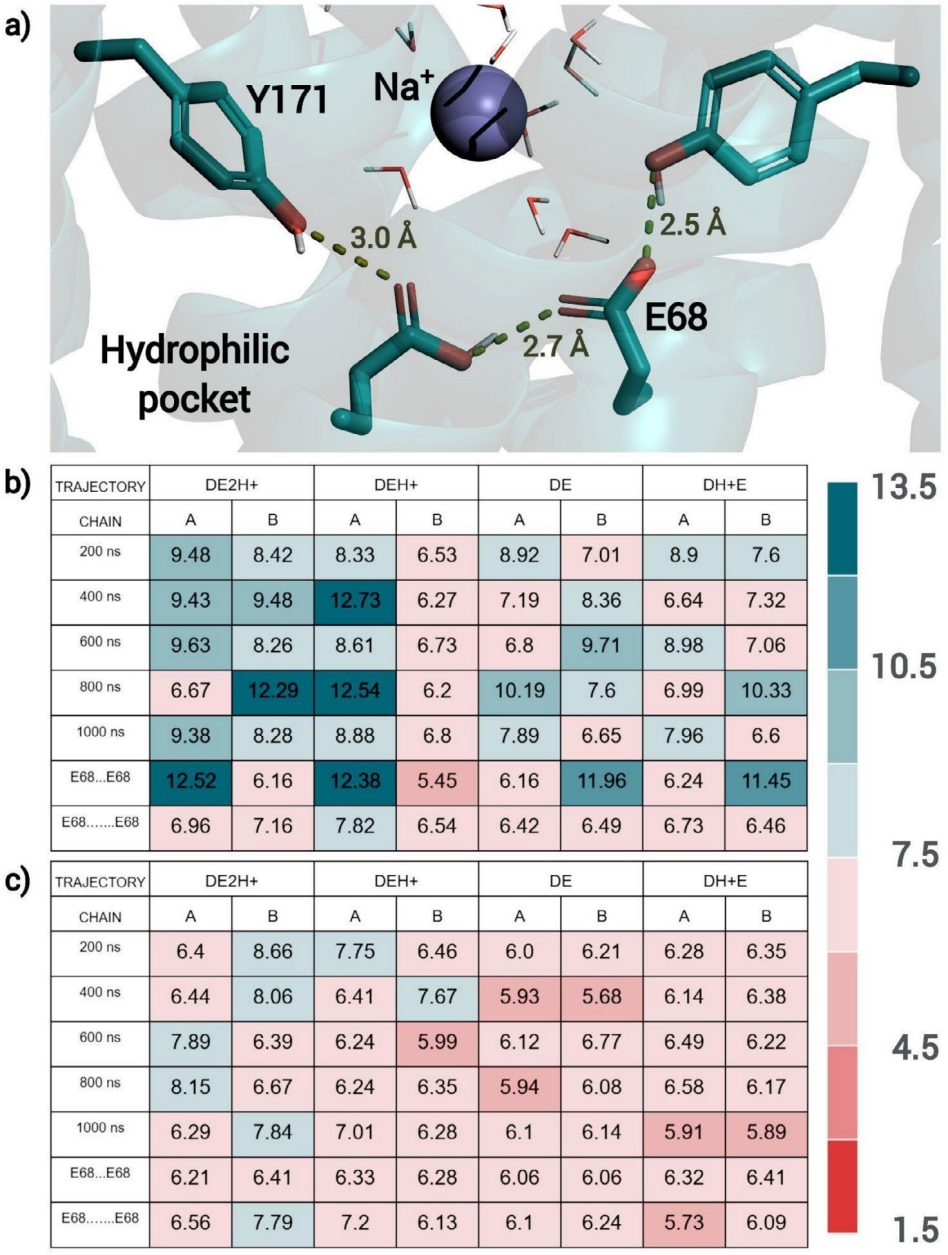
(a) Frame from MD trajectory displaying the hydrogen bonds established between Glu 68 and Tyr 171 residues. Below, p*K*_a_ values of Glu 68 (b) and Asp 39 (c) for configurations extracted from four trajectories with different protonation states (calculated by PROPKA); the frames were taken every 200 ns or were selected for corresponding to the minimal and maximal distance between the Glu 68 pair. The color gradient was used to discriminate between different ranges of p*K*_a_ values.

In view of the crucial part that the highly conserved Asp 39 and Glu 68 residues play in *Eg*VIT1's antiport mechanism through their transient protonation/deprotonation, as described by Kato et al., the behavior of OsVIT2's pore has been further explored via MD simulations, comparing different protonation states of these ionizable residues. As can be seen in Figures 3, S5, and S6 and Table S1, the protonation state of these residues affects the hydration, as well as the conformation of the pore, impacting both the configuration of the backbone and the orientation of the side chains. Beyond that, the hydrophilic pocket showed an ever‐shifting hydrogen bond network between the Glu 68 and Tyr 171 pairs along every trajectory (as can be seen in Figures 4 and S7) [70], as well as a persistent invasion by Na^+^ ions (which had been added for the neutralization of the system) along the trajectories in which at least one of the Glu 68 and neither Asp 39 is ionized; this invasion affected not only the configuration of the hydrophilic pocket itself, but also the distance between the pair of Leu 51, the innermost residues of the hydrophobic seal, which move consistently in orchestration with the pair of Glu 68 along the DEH+ trajectories (as can also be seen in Figures 3, S5 and S6). Ultimately, the protonation state of the Glu 68 pair affects the dynamism of the pore, as evidenced by Figures 3, S5, and S6 and Table S1: while DE2H+ presents very consistent distance values (along trajectories as well as among replicas), DEH+ and DE show some variation, with multimodal distributions and some discrepancy among replicas (Table S1). In particular, DEH+ appears to be very dynamic and susceptible to disturbances.

As for the hydration of the pore, when at least one Glu 68 is protonated, the probability of finding a water molecule at the bottom of the hydrophilic pocket falls compared to the simulations in which the Glu 68 pair is ionized. This can be inspected in the Radial Distribution Function (RDF) plots of Figures 3, S5, and S6, in which *g*(*r*) corresponds to the density of particles (in this case, water molecules) as a function of the distance *r* from a reference atom, relative to the density of an ideal gas. The ionization of the Glu 68 pair makes it not only more hydrophilic but also even more conducive to the above‐mentioned invasion of the hydrophilic pocket by Na^+^ ions, which drag several water molecules along (Figure 3). In fact, the protonation state of these residues seems to modulate the hydropathy of the pocket—making it hydrophilic, depending on the polarity of the glutamate pair—something that might play a part in the transport mechanism. Also, while also affected by the protonation state of the Glu 68 pair, the presence of water molecules near Tyr 171 is consistent along the simulations (Figures 3, S5, and S6).

In spite of these effects associated with the protonation state of the ionizable residues that constitute the hydrophilic pocket on the hydration and conformation of the pore, the hydrophobic seal remained consistently impermeable throughout the MD simulations, as is evidenced in Figures 3, S5, and S6, not only by the distancing plots, but mainly by the RDF plots which show the consistent absence of water molecules in the vicinity of Leu 51, the innermost residue of the hydrophobic seal.

Beyond that, we also observed an extreme variation of calculated p*K*_a_ values for Asp 39 and, particularly, Glu 68 not only between, but also along trajectories (Figure 4)—as an example, along DE2H+ trajectory, the p*K*_a_ value of chain B's Glu 68 residue reached 12.29, as well as 6.16. This extreme variation corroborates not only the association of the Fe^2+^/H^+^ antiport with the transient protonation/deprotonation of Asp 39 and Glu 68 but also suggests a constant fluctuation of protonation states because of conformational changes and ever‐changing electrostatic interactions inside the hydrophilic pocket; in other words, we believe that different protonation states coexist in dynamic equilibrium, with a predominance of DE2H+. It is probable that the consistent presence of water molecules near Tyr 171 plays an important role in this dynamic, enabling the proton transfer.

**FIGURE 5:**
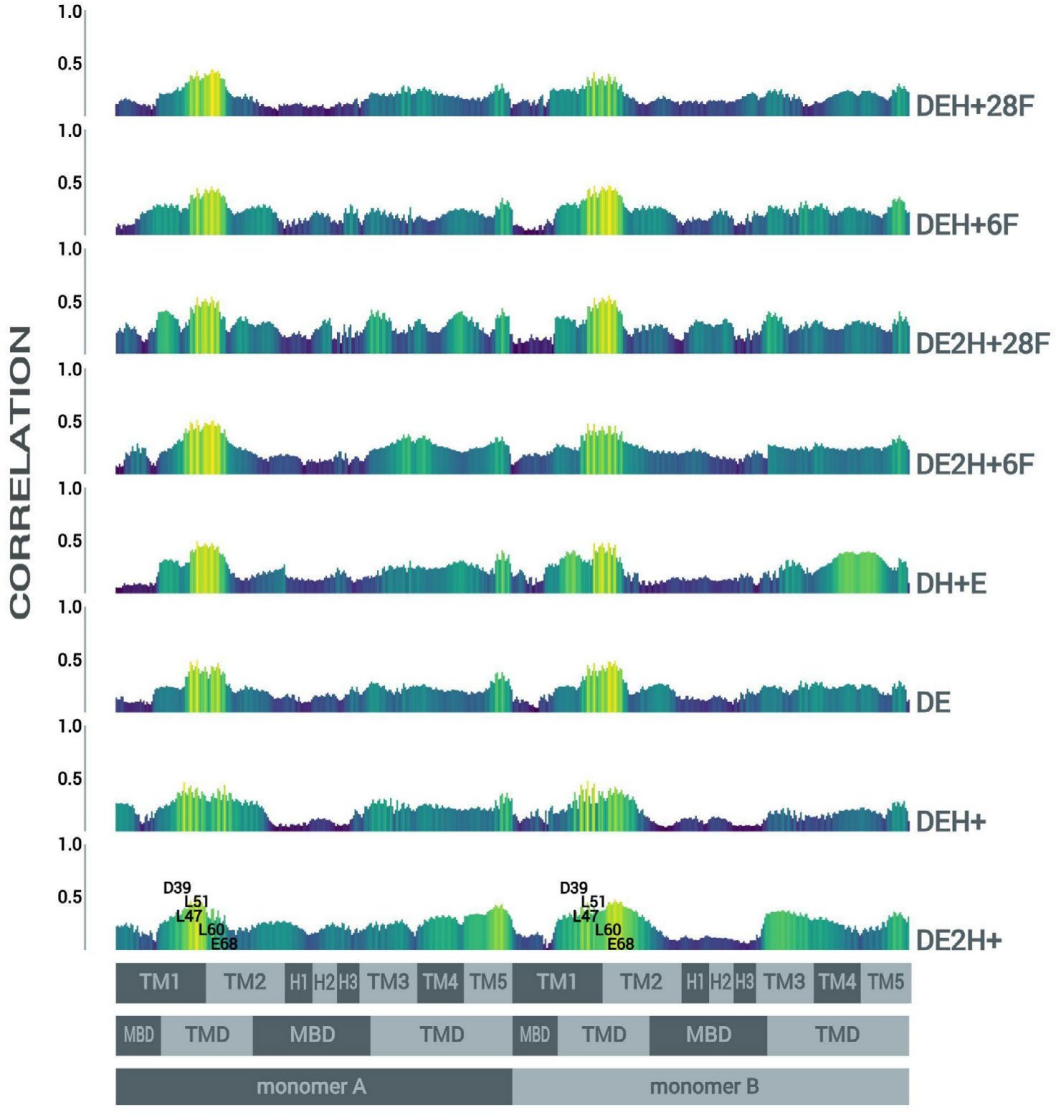
Average cross‐correlation of pairs of Leu 47, 51, and 60, Asp 39 and Glu 68 with every residue down the protein for trajectories (one replica) with different protonation states of the ionizable residues that constitute the hydrophilic pocket (Asp 39 and Glu 68) and occupation states of the MBS. These plots were produced with the R package bio3d.

Finally, when it comes to the integration between the conformational changes observed in the pore and the rest of the protein, we did not observe any significant correlation between the pore residues (i.e., the pairs of bulkier apolar residues that line up the seal plus the pairs of ionizable residues that constitute the hydrophilic pocket) and other residues or regions of the protein—except for a slightly higher correlation with the transmembrane regions, independent of protonation state (Figure 5).

### 3.4 The Iron‐Prospecting Flexible Arms

**FIGURE 6:**
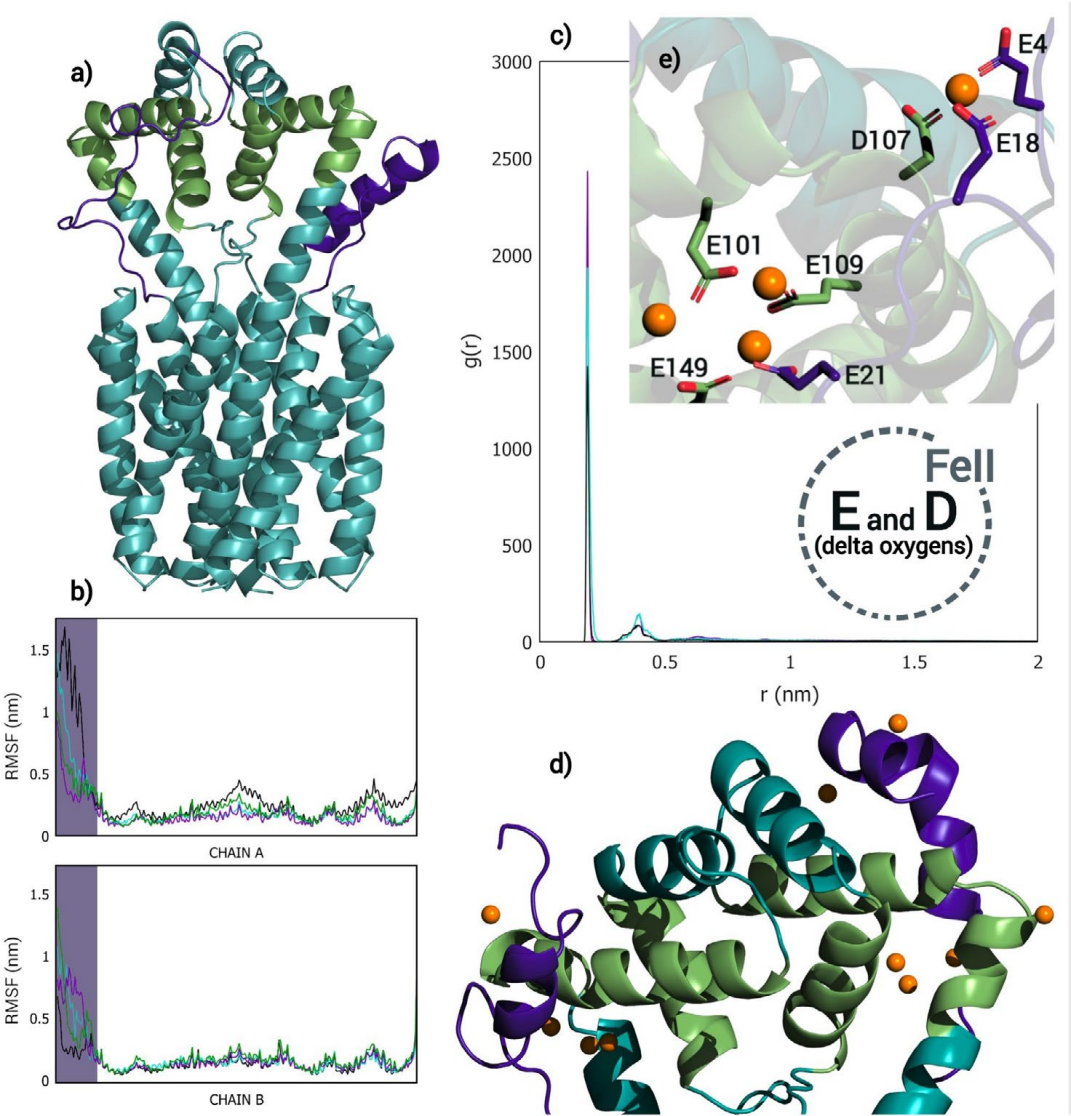
(a) Frame from MD trajectory, to illustrate the prospecting behavior of the flexible arms. (b) RMSF plots for chain A and B; the highlighted regions correspond to the flexible arms and each line color corresponds to a protonation state of the ionizable residues that constitute the hydrophilic pocket (DE2H+ in black; DEH+ in cyan; DE in purple and DH+E in green). (c) RDF plots for the DE2H+28F (black); DEH+28F (cyan) and DE28F (purple) trajectories, showing the probability of finding a Fe^2+^ ion in the vicinity of the Asp and Glu residues carried by the flexible arms, relative to that for an ideal gas. (d) Frame from MD trajectory, to illustrate the interaction between Fe^2+^ ions and the flexible arms. (e) Detail of Glu residues carried by the flexible arms (as well as Asp and Glu residues that constitute the MBS) interacting with Fe^2+^ ions.

Throughout the molecular dynamics simulations, the cytoplasmic portion of TM1 has behaved in accordance with its inherently disorganized nature: unfolding, stretching, and waving (in a way that evokes an inflatable air puppet), while also showing an elevated RMSF (Figure 6). We hypothesize that these “flexible arms” are able to prospect the surroundings of the protein, contributing to the iron capturing function that has already been attributed to the MBD [20], since they carry a series of residues potentially capable of interacting and coordinating with Fe^2+^, like His 19 and 23, Glu 4, 9, 10, 19, and 21, Asp 8 and 17, and Met 1 [71] and keeping in mind that the peptide backbone can also contribute to complexation with metal ions through its carbonyl‐O and amide‐N donor atoms [72]. Also, histidine, glutamate, aspartate, and methionine correspond to ≅30% of the residues that constitute the regions aligned to OsVIT2's flexible arms from the 5000 sequences considered in our alignment (Figure S1). Histidine is particularly enriched, corresponding to 10.6% of the above‐mentioned residues, while its frequency in membrane proteins from plants is 1.94% [73].

Employing MD simulations (systems DE2H + 28F, DEH + 28F, and DE28F, as specified in Table 1), we observed the consistent interaction of Fe^2+^ with the acidic residues carried by the flexible arms, as can be seen in Figure 6. Apart from that, it has also been observed that the occupation of the MBS affects neither the conformation and hydration of the pore nor the correlation between pore residues and the rest of the protein (Figure S8).

### 3.5 Asp39Ala Mutant

**FIGURE 7:**
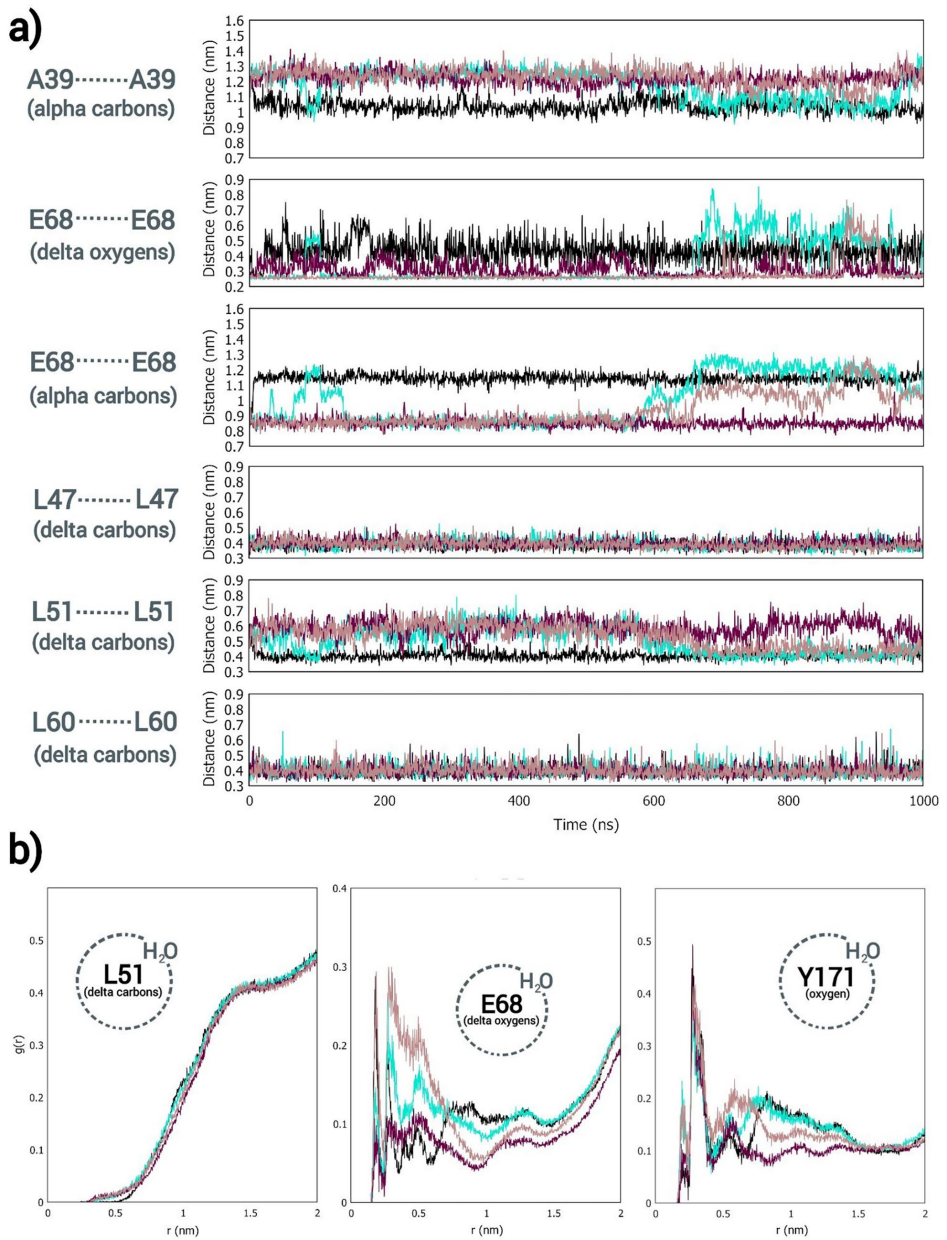
(a) Variation of the distance between pairs of Ala or Asp 39, Glu 68, Leu 47, 51, and 60, along 1 μs long MD simulations, comparing OsVIT2 and its mutant; each color corresponds to a different system (DE2H+ in black; DEH+ in cyan; AE2H+ in dark pink and AEH+ in light pink). (b) RDF plots for the same trajectories, showing the hydration layers of Glu 68 and Tyr 171 but consistent lack of hydration of Leu 51.

The Asp39Ala mutant has been chosen for simulation because the equivalent *Eg*VIT1 mutant had already been functionally characterized by Kato et al., being unable to complement the growth inhibition of a CCC1 knockout yeast strain and presenting diminished transport activity in liposome assays. As can be seen in Figure 7 and Table S1, this alteration impacted the conformation of the pore for both systems simulated (AEH+ and AE2H+): while the alpha carbons of the mutated pair of residues are farther, when compared to the non‐mutated pair, the Glu 68 pair ended up nearer in the Asp39Ala mutant—again, there is a movement correlation between this pair of residues and the Leu 51 pair inside the hydrophobic seal. Beyond the configuration of the pore, the analysis of the trajectories showed no invasion of the hydrophilic pocket by the Na^+^ ions, which could point to the reason behind this mutant's loss of function, namely the transformation of a cation attracting entrance hall by a much less inviting apolar door at the hydrophilic pocket's access.

### 3.6 Antiport Model

**FIGURE 8:**
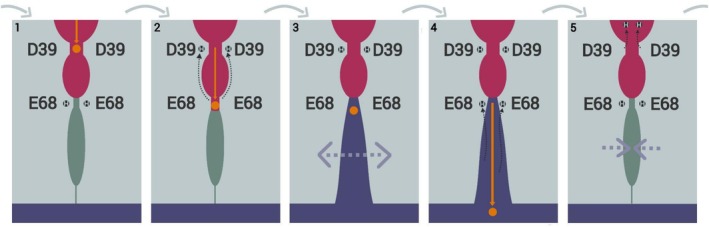
Antiport cycle model. The light grayish blue region represents the protein; the pink area corresponds to the cavity open to the cytoplasm and accessible to its solution; the light green area represents the hydrophobic seal and the purple area corresponds to the acidic vacuolar solution. Fe^2+^ enters the hydrophilic pocket, interacting with the Asp 39 pair and finding both Glu 68 protonated (1). Then, Fe^2+^ displaces the protons (which are momentarily transferred to Asp 39), establishing an interaction with the Glu 68 pair (2). The ion's presence at the bottom of the cavity brings about the opening of the seal and, consequently, its invasion by the acidic vacuolar solution (3). The contact with the acidic vacuolar solution promotes the reprotonation of the Glu 68 pair and the iron's released into the vacuole (4). Finally, the hydrophobic seal recloses and the protons are released to the cytoplasmic solution (5).

Considering the obtained results, we propose an antiport model for OsVIT2 in line with the one proposed by Kato et al. for *Eg*VIT1 (Figure 8): starting from the seemingly prevalent protonation state (with both Glu 68 protonated), Fe^2+^ enters the hydrophilic pocket, interacting with the Asp 39 pair (1). In the sequence, Fe^2+^ displaces the protons (which are momentarily transferred to Asp 39), establishing an interaction with the Glu 68 pair (2). The ion's presence at the bottom of the cavity brings about the opening of the seal and, consequently, its invasion by the acidic vacuolar solution (3). The contact with the acidic vacuolar solution promotes the reprotonation of the Glu 68 pair and the iron's release into the vacuole (4). After that, the hydrophobic seal recloses and the protons are released to the cytoplasmic solution, restoring the system to its original state (5).

## 4 Conclusion

The OsVIT2 model presents its family's idiosyncratic fold and pore architecture. The configuration and hydration of the pore depends on the protonation state of the ionizable residues that constitute the hydrophilic pocket. Conversely, the configuration of the pore affects the p*K*_a_ values of these residues, which fluctuate substantially, indicating that different protonation states coexist in dynamic equilibrium. Apart from that, we observed a shifting hydrogen bond network between the residues, which constitute the hydrophilic pocket. The opening of the pore consists of a very subtle conformational change, showing a rather diffuse integration with the dynamics of the rest of the protein, as well as an orchestration between the pairs of apolar residues that line the hydrophobic seal and the pairs of ionizable residues that constitute the hydrophilic pocket. Additionally, there seems to be an interplay between the pore's conformation and the MBS, suggesting a possible allosteric mechanism. Another noteworthy observation was the behavior of the cytoplasmic portion of TM1: these “flexible arms” unfolded, stretched, and waved throughout the MD simulations, showing an elevated RMSF. Given their interaction with Fe^2+^ ions and their potential for metal coordination, it is possible that they contribute to the iron‐capturing function of the MBD. Such elucidations lay the computational foundations upon which we intend to engineer OsVIT2 and contribute to iron biofortification in rice.

## Author Contributions

**L. B. Arend:** methodology, data curation, validation, investigation, visualization, writing – original draft, writing – review and editing, formal analysis. **D. S. Lima:** methodology, formal analysis, visualization, investigation, data curation. **M. G. S. Costa:** methodology, visualization, formal analysis, investigation. **F. K. Ricachenevsky:** conceptualization, supervision, project administration. **H. Verli:** project administration, supervision, conceptualization, funding acquisition, writing – review and editing, formal analysis, resources, visualization, methodology.

## Peer Review

The peer review history for this article is available at https://www.webofscience.com/api/gateway/wos/peer‐review/10.1002/prot.26843.

## Supporting information


**Table S1.** Average distances (nm) between pairs of ionizable residues that constitute the hydrophilic pocket and pairs of bulkier apolar residues that line the hydrophobic seal for MD trajectories DE2H+ (triplicates), DEH+ (triplicates), DE (triplicates), DH + E (triplicates), AE2H+ and AEH+. Standard deviation values are between parenthesis. When a multimodal distribution was observed, the average distance and standard deviation values for each mode have been indicated, as well as the percentage of the total distribution of each mode.
**Figure S1.** (a) Logo plot contemplating residues 39–76 and residue 171; Asp 39, Leu 47, 51, and 60, Glu 68, Met 76 and Tyr 171 have been indicated with black arrow heads. (b) Logo plot contemplating residues 1–27 (which correspond to the flexible arms); histidine, glutamate, aspartate, and methionine are colored, while the remaining residues are kept black. (c) Pie chart representing the frequency of histidine (10.6%), glutamate (9.2%), aspartate (6.3%), methionine (3.5%), and remaining residues (70.4%) in the regions aligned to OsVIT2’s flexible arms from the 5000 sequences considered in our alignment.
**Figure S2.** (a) PLDDT (predicted local distance difference test) plot for OsVIT2 AlphaFold model, which indicates the local quality of the model; more specifically, this confidence metric corresponds to the percentage of correctly predicted interatomic distances between a residue and all the others within a predetermined radius. This can also be visualized in the OsVIT2 tridimensional model (c), where the residues are colored by their pLDDT values (the colder the color, the higher the confidence); as can be seen, most of the model presents very high confidence, while the cytoplasmic portion of TM1 presents low confidence. (b) PAE (predicted aligned error) plots for OsVIT2 AlphaFold model, which relates to the global quality of the model; more specifically, the PAE corresponds to the error associated with the position of a given residue, when the predicted and real structures are properly aligned in relation to another residue.
**Figure S3.** Jpred4 (a) and PredictProtein (b) results for the cytoplasmic portion of TM1.
**Figure S4.** Superimposition of the extremest configurations assumed by OsVIT2 along the trajectory of atomic displacement generated for normal modes 35 (a), 82 (b), 87 (c), and 97 (d); spheres indicate Leu 47, 51, and 60, Asp 39 and Glu 68.
**Figure S5.** (a) Variation of the distance between pairs of ionizable residues that constitute the hydrophilic pocket and pairs of bulkier apolar residues that line the hydrophobic seal along replica; each line color corresponds to an protonation state of the ionizable residues that constitute the hydrophilic pocket (DE2H+ in black; DEH+ in cyan; DE in purple and DH + E in green). (b) RDF plots for the same replicas, showing the hydration layers of Glu 68 and Tyr 171 but consistent lack of hydration of Leu 51. Replica 2.
**Figure S6.** (a) Variation of the distance between pairs of ionizable residues that constitute the hydrophilic pocket and pairs of bulkier apolar residues that line the hydrophobic seal along replica; each line color corresponds to an protonation state of the ionizable residues that constitute the hydrophilic pocket (DE2H+ in black; DEH+ in cyan; DE in purple and DH + E in green). (b) RDF plots for the same replicas, showing the hydration layers of Glu 68 and Tyr 171 but consistent lack of hydration of Leu 51. Replica 3.
**Figure S7.** p*K*_a_ values, side chain (SC), and backbone (BB) hydrogen bonds and coulombic interactions (CI) established by Glu 68 and Asp 39 for configurations extracted from four trajectories with different protonation states; the frames were taken every 200 ns or were selected for corresponding to the minimal and maximal distance between the Glu 68 pair.
**Figure S8.** Variation of the distance between pairs of ionizable residues that constitute the hydrophilic pocket and pairs of bulkier apolar residues that line the hydrophobic seal along 1 μs long MD simulations for DE2H+ in black, DE2H + 6F in dark pink and DE2H + 28F in light pink (a) and DEH+ in black, DEH + 6F in dark pink and DEH + 28F in light pink (b); each color corresponds to an occupation state of the MBS. (c) Variation of the distance between Glu 68 and the nearest Na + (in the case of DEH + 6F in dark pink) or Fe2+ (in the case of DEH + 28F in light pink). The RDF plots for DE2H+ (black), DE2H + 6F (dark pink) and DE2H + 28F (light pink) (d) and DEH+ (black), DEH + 6F (dark pink), and DEH + 28F (light pink) (e) are also shown.
**Figure S9.** Variation of the potential energy of the systems along 1 μs long MD simulations for DE2H+ replicas (a–c), DEH+ replicas (d–f), DE replicas (g–i), DH + E replicas (j–l), DE2H + 28F (m), DEH + 28F (n), DE28F (o), AE2H+ (p), and AEH+ (q).

## Data Availability

Parameter, topology and structure files, as well as R scripts were made available in the following GitHub repository: https://github.com/laisarend/OsVIT2.

## References

[1] T. Palayullaparambil , A. Krishna , and S. A. Ceasar , “Biofortification of Crops to Fight Anemia: Role of Vacuolar Iron Transporters,” Journal of Agricultural and Food Chemistry 71, no. 8 (2023): 3583–3598. 36802625 10.1021/acs.jafc.2c07727

[2] World Health Organization , Iron Deficiency Anemia Assessment Prevention and Control: A Guide for Program Managers (WHO, 2001).

[3] K. Shubham , T. Anukiruthika , S. Dutta , A. V. Kashyap , J. A. Moses , and C. Anandharamakrishnan , “Iron Deficiency Anemia: A Comprehensive Review on Iron Absorption, Bioavailability and Emerging Food Fortification Approaches,” Trends in Food Science and Technology 99 (2020): 58–75.

[4] N. Abbaspour , R. Hurrell , and R. Kelishadi , “Review on Iron and Its Importance for Human Health,” Journal of Research in Medical Sciences: The Official Journal of Isfahan University of Medical Sciences 19, no. 2 (2014): 164–174. 24778671 PMC3999603

[5] M. I. Gómez , C. B. Barrett , T. Raney , et al., “Post‐Green Revolution Food Systems and the Triple Burden of Malnutrition,” Food Policy 42 (2013): 129–138.

[6] R. D. Graham , M. Knez , and R. M. Welch , “How Much Nutritional Iron Deficiency in Humans Globally Is due to an Underlying Zinc Deficiency?,” in Advances in Agronomy, vol. 115 (Elsevier Inc., 2012), 1–40.

[7] R. A. Sperotto , “Zn/Fe Remobilization From Vegetative Tissues to Rice Seeds: Should I Stay or Should I Go? Ask Zn/Fe Supply!,” Frontiers in Plant Science 4 (2013): 1–4. 24294216 10.3389/fpls.2013.00464 PMC3827551

[8] K. Bashir , Y. Ishimaru , and N. K. Nishizawa , “Iron Uptake and Loading Into Rice Grains,” Rice 3, no. 2–3 (2010): 122–130.

[9] R. S. dos Santos , A. T. De Araujo , C. Pegoraro , and A. C. de Oliveira , “Dealing With Iron Metabolism in Rice: From Breeding for Stress Tolerance to Biofortification,” Genetics and Molecular Biology 40, no. 1 (2017): 312–325. 28304072 10.1590/1678-4685-GMB-2016-0036 PMC5452141

[10] S. A. Kim , T. Punshon , A. Lanzirotti , et al., “Localization of Iron in Arabidopsis Seed Requires the Vacuolar Membrane Transporter VIT1,” Science 314 (2006): 1295–1298. 17082420 10.1126/science.1132563

[11] S. A. Kim and M. L. Guerinot , “Mining Iron: Iron Uptake and Transport in Plants,” FEBS Letters 581, no. 12 (2007): 2273–2280. 17485078 10.1016/j.febslet.2007.04.043

[12] K. Slavic , S. Krishna , A. Lahree , et al., “A Vacuolar Iron‐Transporter Homologue Acts as a Detoxifier in Plasmodium,” Nature Communications 7, no. 1 (2016): 10403, 10.1038/ncomms10403. PMC4735874 26786069

[13] H. Puren , B. J. Reddy , A. Sarma , S. K. Singh , and W. A. Ansari , “Molecular Approaches for Biofortification of Cereal Crops,” in Biofortification in Cereals: Progress and Prospects (Springer Nature Singapore Pte Ltd, 2023), 21–58.

[14] J. M. Connorton and J. Balk , “Iron Biofortification of Staple Crops: Lessons and Challenges in Plant Genetics,” Plant & Cell Physiology 60, no. 7 (2019): 1447–1456. 31058958 10.1093/pcp/pcz079 PMC6619672

[15] T. P. A. Krishna , T. Maharajan , and S. A. Ceasar , “The Role of Membrane Transporters in the Biofortification of Zinc and Iron in Plants,” Biological Trace Element Research 201, no. 1 (2023): 464–478. 35182385 10.1007/s12011-022-03159-w

[16] K. Bashir , R. Takahashi , S. Akhtar , Y. Ishimaru , H. Nakanishi , and N. K. Nishizawa , “The Knockdown of OsVIT2 and MIT Affects Iron Localization in Rice Seed,” Rice 6, no. 1 (2013): 31–36. 24280309 10.1186/1939-8433-6-31 PMC4883708

[17] J. Che , N. Yamaji , and J. F. Ma , “Role of a Vacuolar Iron Transporter OsVIT2 in the Distribution of Iron to Rice Grains,” New Phytologist 230, no. 3 (2021): 1049–1062. 33474769 10.1111/nph.17219

[18] Y. Zhang , Y. H. Xu , H. Y. Yi , and J. M. Gong , “Vacuolar Membrane Transporters OsVIT1 and OsVIT2 Modulate Iron Translocation Between Flag Leaves and Seeds in Rice,” Plant Journal 72, no. 3 (2012): 400–410. 10.1111/j.1365-313X.2012.05088.x 22731699

[19] P. Labarbuta , K. Duckett , C. H. Botting , et al., “Recombinant Vacuolar Iron Transporter Family Homologue PfVIT From Human Malaria‐Causing Plasmodium Falciparum Is a Fe2+/H+ Exchanger,” Scientific Reports 7, no. 1 (2017): 42850, 10.1038/srep42850. 28198449 PMC5309874

[20] T. Kato , K. Kumazaki , M. Wada , et al., “Crystal Structure of Plant Vacuolar Iron Transporter VIT1,” Nature Plants 5, no. 3 (2019): 308–315. 30742036 10.1038/s41477-019-0367-2

[21] J. A. McCammon , B. R. Gelin , and M. Karplus , “Dynamics of Folded Proteins,” Nature 267, no. 5612 (1977): 585–590. 301613 10.1038/267585a0

[22] S. A. Hollingsworth and R. O. Dror , “Molecular Dynamics Simulation for All,” Neuron 99, no. 6 (2018): 1129–1143. 30236283 10.1016/j.neuron.2018.08.011 PMC6209097

[23] M. Karplus and J. A. McCammon , “Molecular Dynamics Simulations of Biomolecules,” Nature Structural Biology 9, no. 9 (2002): 646–652. 12198485 10.1038/nsb0902-646

[24] A. Hospital , J. R. Goñi , M. Orozco , and J. L. Gelpí , “Molecular Dynamics Simulations: Advances and Applications,” Advances in Applied Bioinformatics and Chemistry 8, no. 1 (2015): 37–47. 10.2147/AABC.S70333 PMC4655909 26604800

[25] K. Goossens and H. De Winter , “Molecular Dynamics Simulations of Membrane Proteins: An Overview,” Journal of Chemical Information and Modeling 58, no. 11 (2018): 2193–2202. 30336018 10.1021/acs.jcim.8b00639

[26] M. G. S. Costa , P. R. Batista , P. M. Bisch , and D. Perahia , “Exploring Free Energy Landscapes of Large Conformational Changes: Molecular Dynamics With Excited Normal Modes,” Journal of Chemical Theory and Computation 11, no. 6 (2015): 2755–2767. 26575568 10.1021/acs.jctc.5b00003

[27] I. Bahar , T. R. Lezon , A. Bakan , and I. H. Shrivastava , “Normal Mode Analysis of Biomolecular Structures: Functional Mechanisms of Membrane Proteins,” Chemical Reviews 110, no. 3 (2010): 1463–1497. 19785456 10.1021/cr900095e PMC2836427

[28] J. A. Bauer and V. Bauerová‐Hlinková , “Normal Mode Analysis: A Tool for Better Understanding Protein Flexibility and Dynamics With Application to Homology Models,” in Homology Molecular Modeling—Perspectives and Applications (IntechOpen, 2021), 1–18.

[29] E. J. Haddadian , M. H. Cheng , R. D. Coalson , Y. Xu , and P. Tang , “In Silico Models for the Human Α4β2 Nicotinic Acetylcholine Receptor,” Journal of Physical Chemistry B 112, no. 44 (2008): 13981–13990. 18847252 10.1021/jp804868s PMC2775927

[30] H. Valadié , J. J. Lacapčre , Y. H. Sanejouand , and C. Etchebest , “Dynamical Properties of the MscL of *Escherichia Coli* : A Normal Mode Analysis,” Journal of Molecular Biology 332, no. 3 (2003): 657–674. 12963374 10.1016/s0022-2836(03)00851-9

[31] A. Mickiewicz , J. Sarzyńska , M. Miłostan , et al., “Modeling of the Catalytic Core of *Arabidopsis Thaliana* Dicer‐ Like 4 Protein and Its Complex With Double‐Stranded RNA,” Computational Biology and Chemistry 66 (2017): 44–56. 27907832 10.1016/j.compbiolchem.2016.11.003

[32] O. Kurkcuoglu and P. A. Bates , “Mechanism of Cohesin Loading Onto Chromosomes: A Conformational Dynamics Study,” Biophysical Journal 99, no. 4 (2010): 1212–1220. 20713005 10.1016/j.bpj.2010.06.006 PMC2920725

[33] E. C. Dykeman and O. F. Sankey , “Normal Mode Analysis and Applications in Biological Physics,” Journal of Physics. Condensed Matter 22, no. 42 (2010): 423202, 10.1088/0953-8984/22/42/423202. 21403307

[34] V. Alexandrov , “Normal Modes for Predicting Protein Motions: A Comprehensive Database Assessment and Associated Web Tool,” Protein Science 14, no. 3 (2005): 633–643. 15722444 10.1110/ps.04882105 PMC2279292

[35] K. Hinsen , “Normal Mode Theory and Harmonic Potential Approximations,” in Normal Mode Analysis: Theory and Applications to Biological and Chemical Systems (Chapman and Hall/CRC, 2005), 1–18.

[36] J. Jumper , R. Evans , A. Pritzel , et al., “Highly Accurate Protein Structure Prediction With AlphaFold,” Nature 596, no. 7873 (2021): 583–589. 34265844 10.1038/s41586-021-03819-2 PMC8371605

[37] M. Mirdita , K. Schütze , Y. Moriwaki , L. Heo , S. Ovchinnikov , and M. Steinegger , “ColabFold: Making Protein Folding Accessible to All,” Nature Methods 19, no. 6 (2022): 679–682. 35637307 10.1038/s41592-022-01488-1 PMC9184281

[38] A. Drozdetskiy , C. Cole , J. Procter , and G. J. Barton , “JPred4: A Protein Secondary Structure Prediction Server,” Nucleic Acids Research 43, no. W1 (2015): W389–W394. 25883141 10.1093/nar/gkv332 PMC4489285

[39] M. Bernhofer , C. Dallago , T. Karl , et al., “PredictProtein—Predicting Protein Structure and Function for 29 Years,” Nucleic Acids Research 49, no. W1 (2021): W535–W540. 33999203 10.1093/nar/gkab354 PMC8265159

[40] H. Li , A. D. Robertson , and J. H. Jensen , “Very Fast Empirical Prediction and Rationalization of Protein PK a Values,” Proteins: Structure, Function, and Genetics 61, no. 4 (2005): 704–721. 10.1002/prot.20660 16231289

[41] S. Jo , T. Kim , V. G. Iyer , and W. Im , “CHARMM‐GUI: A Web‐Based Graphical User Interface for CHARMM,” Journal of Computational Chemistry 29, no. 11 (2008): 1859–1865. 18351591 10.1002/jcc.20945

[42] E. L. Wu , X. Cheng , S. Jo , et al., “CHARMM‐GUI Membrane Builder Toward Realistic Biological Membrane Simulations,” Journal of Computational Chemistry 35, no. 27 (2014): 1997–2004. 25130509 10.1002/jcc.23702 PMC4165794

[43] J. Lee , X. Cheng , J. M. Swails , et al., “CHARMM‐GUI Input Generator for NAMD, GROMACS, AMBER, OpenMM, and CHARMM/OpenMM Simulations Using the CHARMM36 Additive Force Field,” Journal of Chemical Theory and Computation 12, no. 1 (2016): 405–413. 26631602 10.1021/acs.jctc.5b00935 PMC4712441

[44] J. Y. Zhou , D. L. Hao , and G. Z. Yang , “Regulation of Cytosolic PH: The Contributions of Plant Plasma Membrane H+‐Atpases and Multiple Transporters,” International Journal of Molecular Sciences 22, no. 23 (2021): 12998. 34884802 10.3390/ijms222312998 PMC8657649

[45] K. Y. Kulichikhin , O. Aitio , T. V. Chirkova , and K. V. Fagerstedt , “Effect of Oxygen Concentration on Intracellular PH, Glucose‐6‐Phosphate and NTP Content in Rice [*Oryza Sativa*] and Wheat [*Triticum Aestivum*] Root Tips: In Vivo 31P‐NMR Study,” Physiologia Plantarum 129, no. 3 (2007): 507–518.

[46] B. B. Buchanan , W. Gruissem , and R. L. Jones , eds., Biochemistry & Molecular Biology of Plants (John Wiley & Sons, Ltd, 2015).

[47] D. Van Der Spoel , E. Lindahl , B. Hess , G. Groenhof , A. E. Mark , and H. J. C. Berendsen , “GROMACS: Fast, Flexible, and Free,” Journal of Computational Chemistry 26, no. 16 (2005): 1701–1718. 16211538 10.1002/jcc.20291

[48] M. J. Abraham , T. Murtola , R. Schulz , et al., “Gromacs: High Performance Molecular Simulations Through Multi‐Level Parallelism From Laptops to Supercomputers,” SoftwareX 1–2 (2015): 19–25.

[49] R. B. Best , X. Zhu , J. Shim , et al., “Optimization of the Additive CHARMM All‐Atom Protein Force Field Targeting Improved Sampling of the Backbone φ, ψ and Side‐Chain Χ1 and Χ2 Dihedral Angles,” Journal of Chemical Theory and Computation 8, no. 9 (2012): 3257–3273. 23341755 10.1021/ct300400x PMC3549273

[50] J. Huang and A. D. Mackerell , “CHARMM36 All‐Atom Additive Protein Force Field: Validation Based on Comparison to NMR Data,” Journal of Computational Chemistry 34, no. 25 (2013): 2135–2145. 23832629 10.1002/jcc.23354 PMC3800559

[51] L. Pravda , D. Sehnal , D. Toušek , et al., “MOLEonline: A Web‐Based Tool for Analyzing Channels, Tunnels and Pores (2018 Update),” Nucleic Acids Research 46, no. W1 (2018): W368–W373. 29718451 10.1093/nar/gky309 PMC6030847

[52] Y. Won , “Force Field for Monovalent, Divalent, and Trivalent Cations Developed Under the Solvent Boundary Potential,” Journal of Physical Chemistry A 116, no. 47 (2012): 11763–11767. 23102428 10.1021/jp309150r

[53] B. J. Grant , A. P. C. Rodrigues , K. M. ElSawy , J. A. McCammon , and L. S. D. Caves , “Bio3d: An R Package for the Comparative Analysis of Protein Structures,” Bioinformatics 22, no. 21 (2006): 2695–2696. 16940322 10.1093/bioinformatics/btl461

[54] B. J. Grant , L. Skjærven , and X. Q. Yao , “The Bio3D Packages for Structural Bioinformatics,” Protein Science 30, no. 1 (2021): 20–30. 32734663 10.1002/pro.3923 PMC7737766

[55] S. Hayward , A. Kitao , and N. Gō , “Harmonicity and Anharmonicity in Protein Dynamics: A Normal Mode Analysis and Principal Component Analysis,” Proteins: Structure, Function, and Bioinformatics 23, no. 2 (1995): 177–186. 10.1002/prot.340230207 8592699

[56] D. A. Case , “Normal Mode Analysis of Protein Dynamics,” Current Opinion in Structural Biology 4, no. 2 (1994): 285–290.

[57] H. Wako and S. Endo , “Normal Mode Analysis as a Method to Derive Protein Dynamics Information From the Protein Data Bank,” Biophysical Reviews 9, no. 6 (2017): 877–893. 29103094 10.1007/s12551-017-0330-2 PMC5711701

[58] L. Skjaerven , S. M. Hollup , and N. Reuter , “Normal Mode Analysis for Proteins,” Journal of Molecular Structure: THEOCHEM 898, no. 1–3 (2009): 42–48.

[59] S. Hayward and B. L. Groot , “Normal Modes and Essential Dynamics,” in Molecular Modeling of Proteins, Methods in Molecular Biology, vol. 443 (Humana Press, 2008), 89–106. 18446283 10.1007/978-1-59745-177-2_5

[60] J. A. Bauer , J. Pavlovíc , and V. Bauerová‐Hlinková , “Normal Mode Analysis as a Routine Part of a Structural Investigation,” Molecules 24, no. 18 (2019): 3293. 31510014 10.3390/molecules24183293 PMC6767145

[61] M. D. Polêto and J. A. Lemkul , “Integration of Experimental Data and Use of Automated Fitting Methods in Developing Protein Force Fields,” Communications Chemistry 5, no. 1 (2022): 38. 35382231 10.1038/s42004-022-00653-z PMC8979544

[62] L. Scrucca , C. Fraley , T. B. Murphy , and A. E. Raftery , Model‐Based Clustering, Classification, and Density Estimation Using Mclust in R, 1st ed. (Chapman and Hall/CRC, 2023).

[63] S. Kumar , K. Tamura , and M. Nei , “Molecular Evolutionary Genetics Analysis Software for Microcomputers,” Computer Applications in the Biosciences 10 (1994): 189–191. 8019868 10.1093/bioinformatics/10.2.189

[64] M. A. Larkin , G. Blackshields , N. P. Brown , et al., “Clustal W and Clustal X Version 2.0,” Bioinformatics 23 (2007): 2947–2948. 17846036 10.1093/bioinformatics/btm404

[65] G. E. Crooks , G. Hon , J. M. Chandonia , and S. E. Brenner , “WebLogo: A Sequence Logo Generator,” Genome Research 14 (2004): 1188–1190. 15173120 10.1101/gr.849004 PMC419797

[66] A. Kelly and K. M. Knowles , eds., Crystallography and Crystal Defects, 2nd ed. (Wiley, 2012).

[67] S. T. Breviglieri , E. T. G. Cavalheiro , and G. O. Chierice , “Correlation Between Ionic Radius and Thermal Decomposition of Fe (II), Co (II), Ni (II), Cu (II) and Zn (II) Diethanoldithiocarbamates,” Thermochimica Acta 356, no. 1‐2 (2000): 79–84, 10.1016/s0040-6031(00)00465-2.

[68] P. Schatzberg , “On the Molecular Diameter of Water From Solubility and Diffusion Measurements,” Journal of Physical Chemistry 71, no. 13 (1967): 4569–4570.

[69] J. S. D'Arrigo , “Screening of Membrane Surface Charges by Divalent Cations: An Atomic Representation,” American Journal of Physiology. Cell Physiology 235, no. 3 (1978): C109–C117. 10.1152/ajpcell.1978.235.3.C109 696813

[70] D. Herschlag and M. Pinney , “Hydrogen Bonds: Simple After All?,” Biochemistry 57, no. 24 (2018): 3338–3352. 29678112 10.1021/acs.biochem.8b00217

[71] I. Dokmanić , M. Sikić , and S. Tomić , “Metals in Proteins: Correlation Between the Metal‐Ion Type, Coordination Number and the Amino‐Acid Residues Involved in the Coordination,” Acta Crystallographica, Section D: Biological Crystallography 64, no. Pt 3 (2008): 257–263. 18323620 10.1107/S090744490706595X

[72] I. Sóvágó , C. Kállay , and K. Várnagy , “Peptides as Complexing Agents: Factors Influencing the Structure and Thermodynamic Stability of Peptide Complexes,” Coordination Chemistry Reviews 256 (2012): 2225–2233.

[73] R. K. Gaur , “Amino Acid Frequency Distribution Among Eukaryotic Proteins,” IIOAB Journal 5, no. 2 (2014): 6–11.

