# Supplementary material for: Molecular Basis for Vacuolar Iron Transport by OsVIT2, a Target for Iron Biofortification in Rice
Source: Proteins. 2025 May 15;93(10):1717–31. doi: 10.1002/prot.26843 (PMC12433260; doi:10.1002/prot.26843)
Supplement: Supplementary file 1 — Table S1. Average distances (nm) between pairs of ionizable residues that constitute the hydrophilic pocket and pairs of bulkier apolar residues that line the hydrophobic seal for MD trajectories DE2H+ (triplicates), DEH+ (triplicates), DE (triplicates), DH + E (triplicates), AE2H+ and AEH+. Standard deviation values are between parenthesis. When a multimodal distribution was observed, the average distance and standard deviation values for each mode have been indicated, as well as the percentage of the total distribution of each mode. Figure S1. (a) Logo plot contemplating residues 39–76 and residue 171; Asp 39, Leu 47, 51, and 60, Glu 68, Met 76 and Tyr 171 have been indicated with black arrow heads. (b) Logo plot contemplating residues 1–27 (which correspond to the flexible arms); histidine, glutamate, aspartate, and methionine are colored, while the remaining residues are kept black. (c) Pie chart representing the frequency of histidine (10.6%), glutamate (9.2%), aspartate (6.3%), methionine (3.5%), and remaining residues (70.4%) in the regions aligned to OsVIT2’s flexible arms from the 5000 sequences considered in our alignment. Figure S2. (a) PLDDT (predicted local distance difference test) plot for OsVIT2 AlphaFold model, which indicates the local quality of the model; more specifically, this confidence metric corresponds to the percentage of correctly predicted interatomic distances between a residue and all the others within a predetermined radius. This can also be visualized in the OsVIT2 tridimensional model (c), where the residues are colored by their pLDDT values (the colder the color, the higher the confidence); as can be seen, most of the model presents very high confidence, while the cytoplasmic portion of TM1 presents low confidence. (b) PAE (predicted aligned error) plots for OsVIT2 AlphaFold model, which relates to the global quality of the model; more specifically, the PAE corresponds to the error associated with the position of a given residue, w [file PROT-93-1717-s001.docx]

Supplementary material


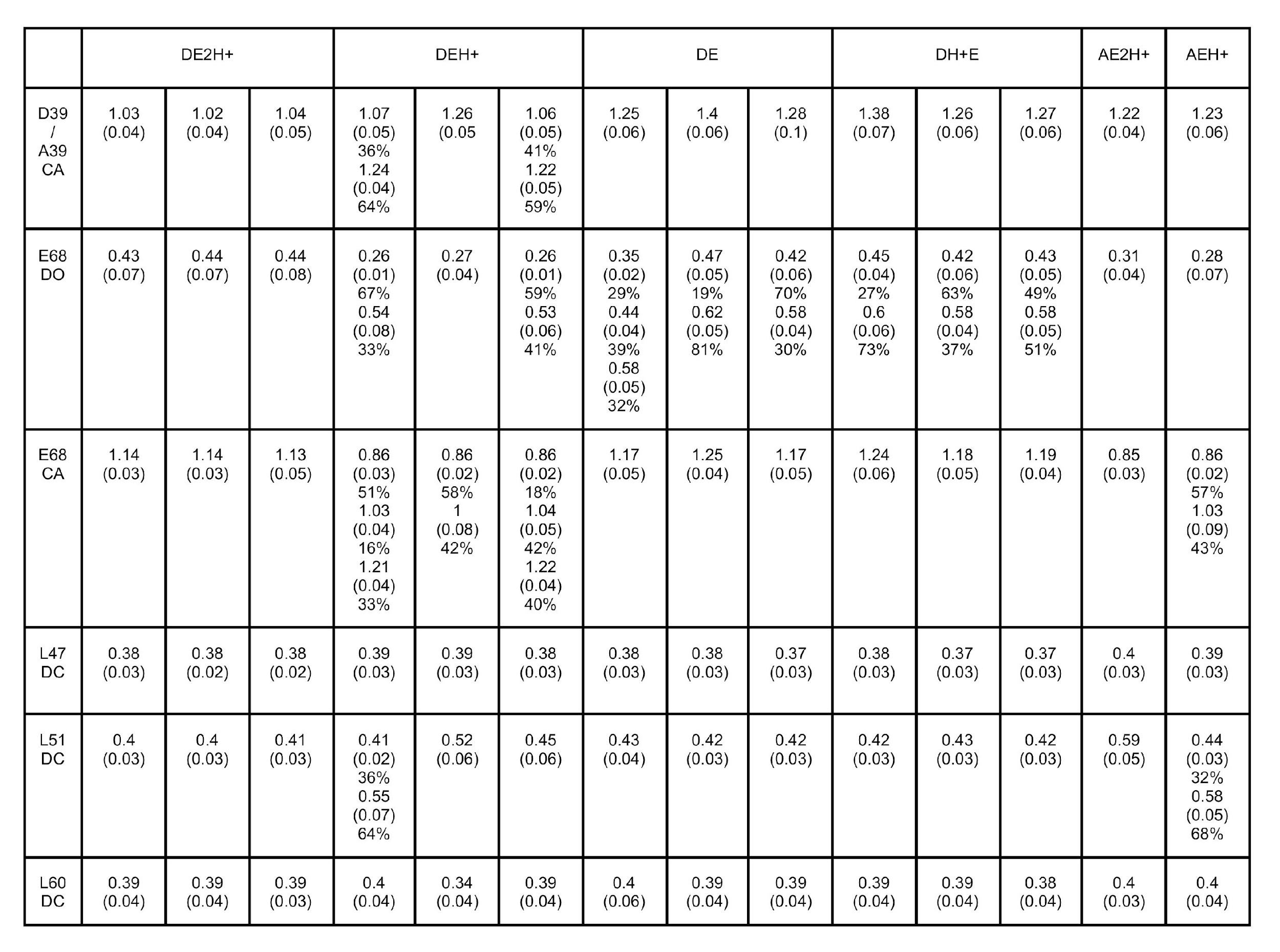


Supplemental Table 1. Average distances (nm) between pairs of ionizable residues that constitute the hydrophilic pocket and pairs of bulkier apolar residues that line the hydrophobic seal for MD trajectories DE2H+ (triplicates), DEH+ (triplicates), DE (triplicates), DH+E (triplicates), AE2H+ and AEH+. Standard deviation values are between parenthesis. When a multimodal distribution was observed, the average distance and standard deviation values for each mode have been indicated, as well as the percentage of the total distribution of each mode.


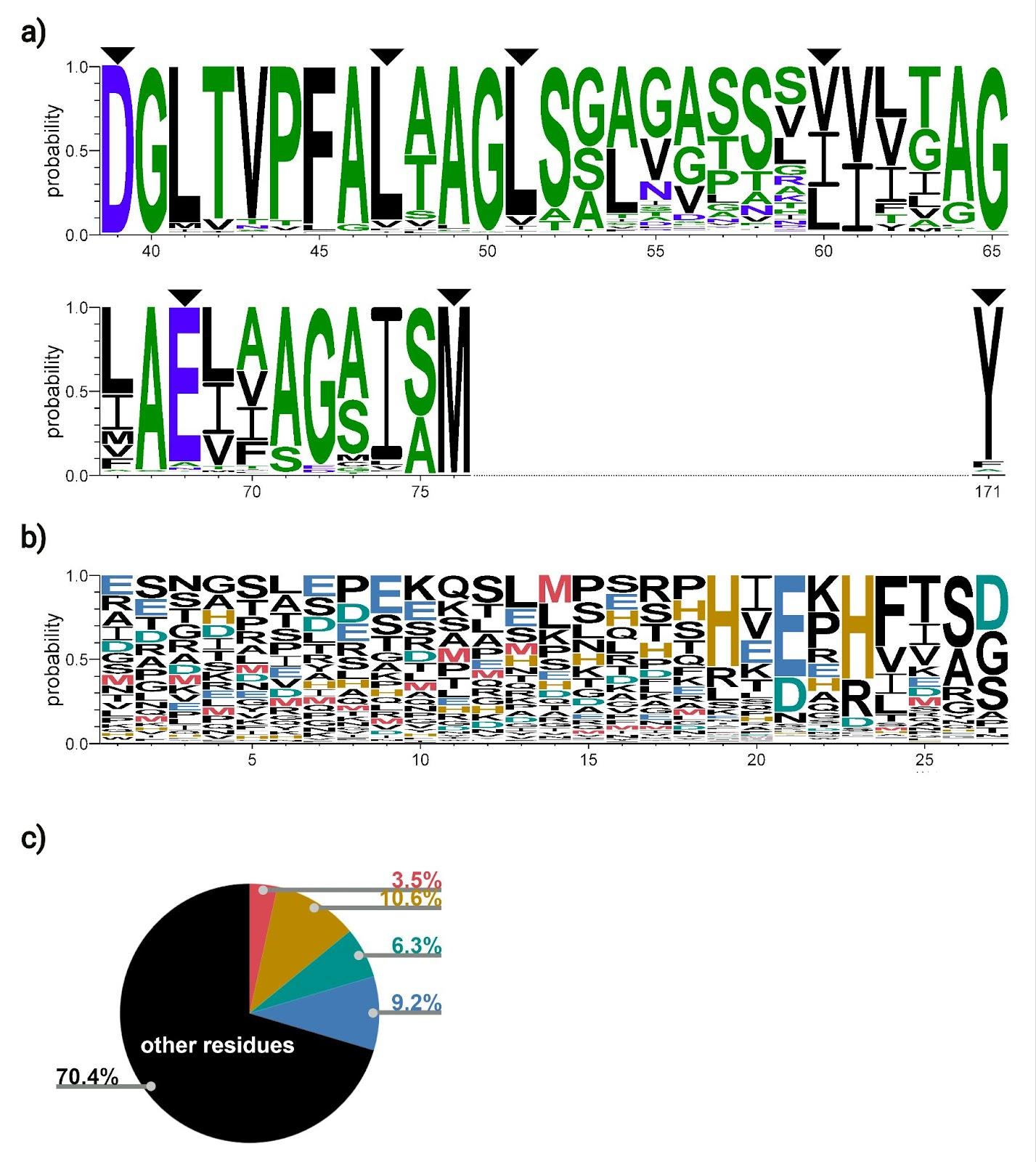


Supplemental figure 1. a) Logo plot contemplating residues 39 to 76 and residue 171; Asp 39, Leu 47, 51 and 60, Glu 68, Met 76 and Tyr 171 have been indicated with black arrow heads. b) Logo plot contemplating residues 1 to 27 (which correspond to the flexible arms); histidine, glutamate, aspartate and methionine are colored, while the remaining residues are kept black. c) Pie chart representing the frequency of histidine (10.6%), glutamate (9.2%), aspartate (6.3%), methionine (3.5%) and remaining residues (70.4%) in the regions aligned to OsVIT2’s flexible arms from the 5000 sequences considered in our alignment.


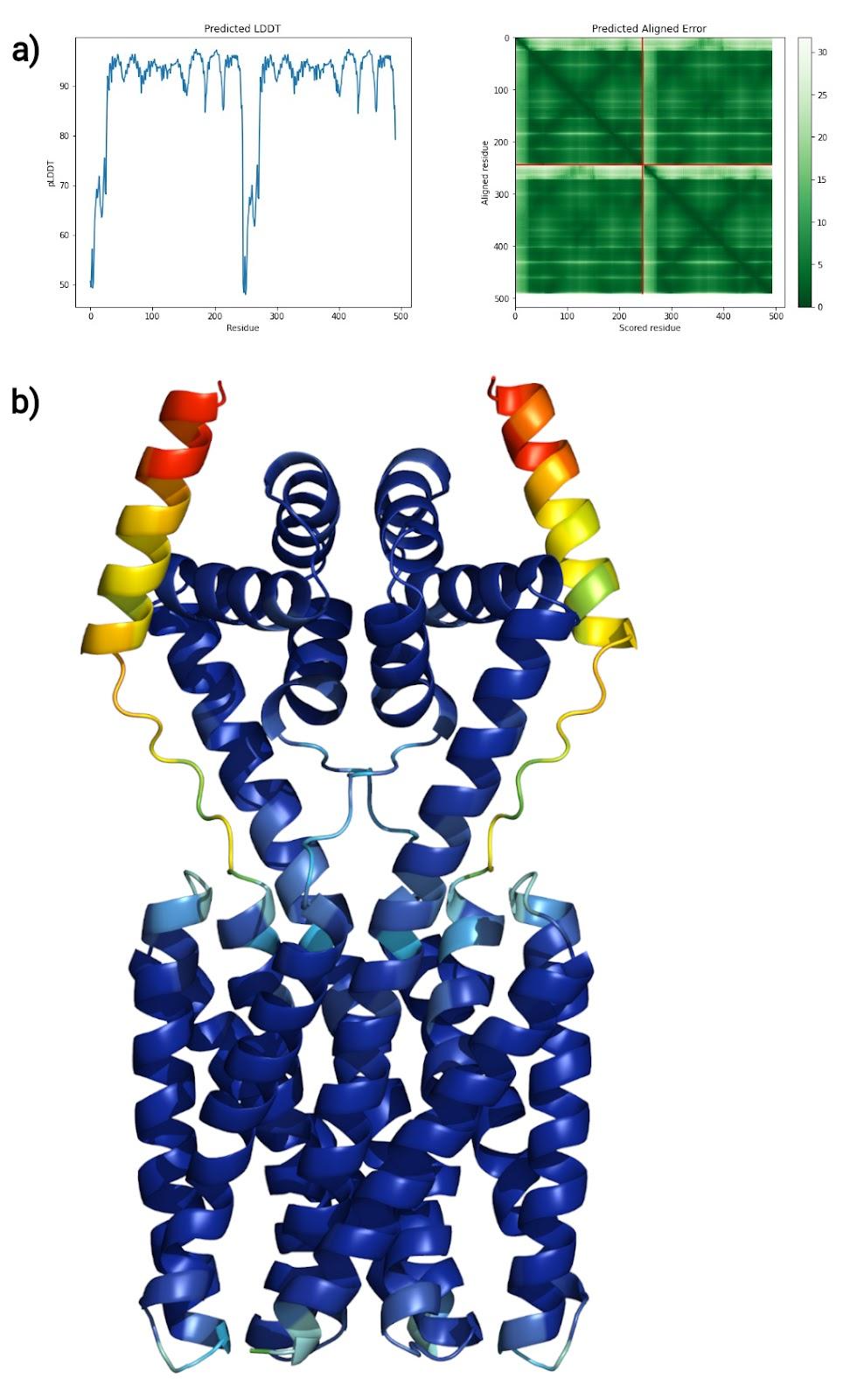


Supplemental Figure 2. a) PLDDT (predicted local distance difference test) plot for OsVIT2 AlphaFold model, which indicates the local quality of the model; more specifically, this confidence metric corresponds to the percentage of correctly predicted interatomic distances between a residue and all the others within a predetermined radius. This can also be visualized in the OsVIT2 tridimensional model (c), where the residues are colored by their pLDDT values (the colder the color, the higher the confidence); as can be seen, most of the model presents very high confidence, while the cytoplasmic portion of TM1 presents low confidence. b) PAE (predicted aligned error) plots for OsVIT2 AlphaFold model, which relates to the global quality of the model; more specifically, the PAE corresponds to the error associated with the position of a given residue, when the predicted and real structures are properly aligned in relation to another residue.


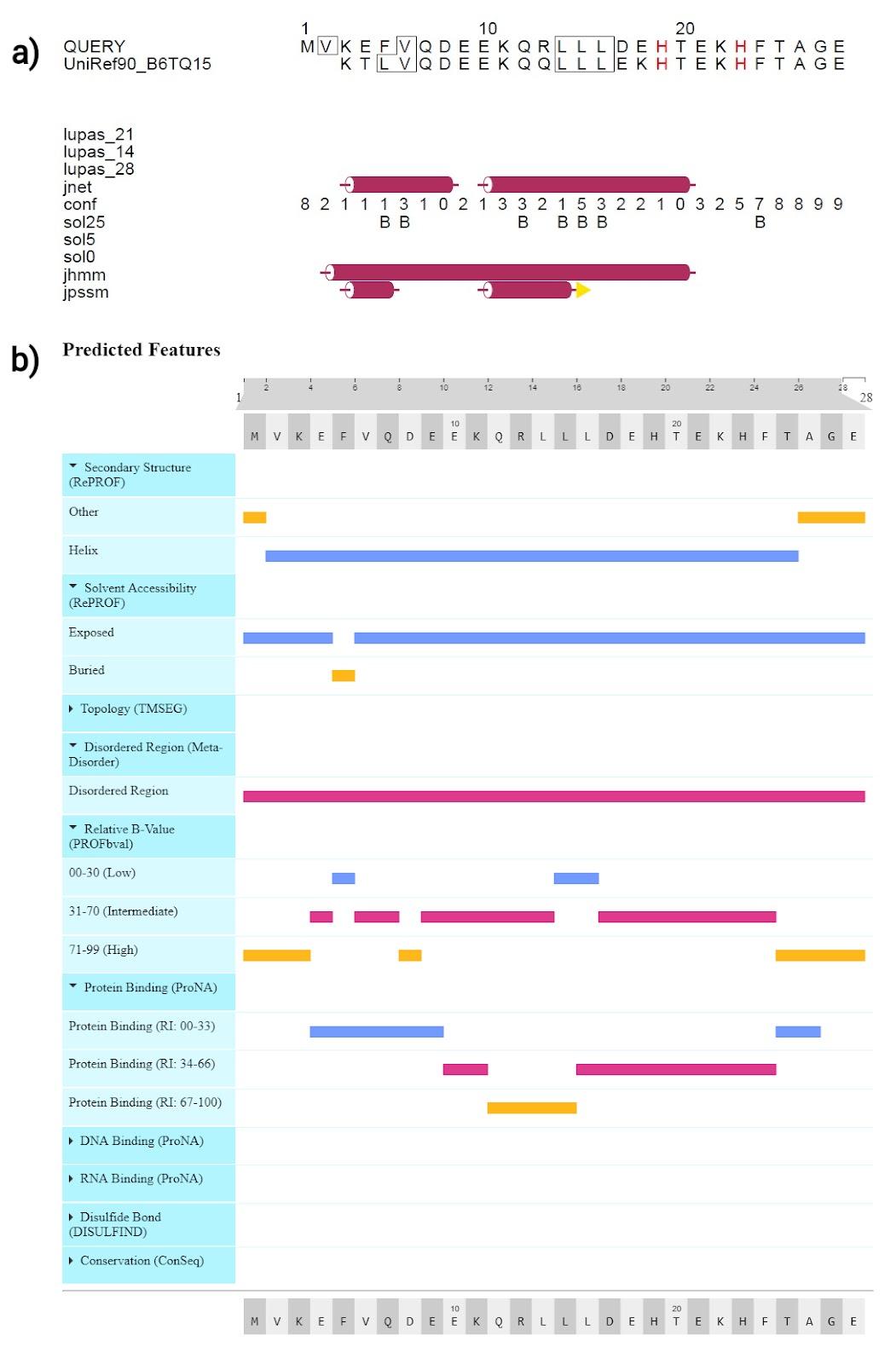


Supplemental Figure 3. Jpred4 (a) and PredictProtein (b) results for the cytoplasmic portion of TM1.


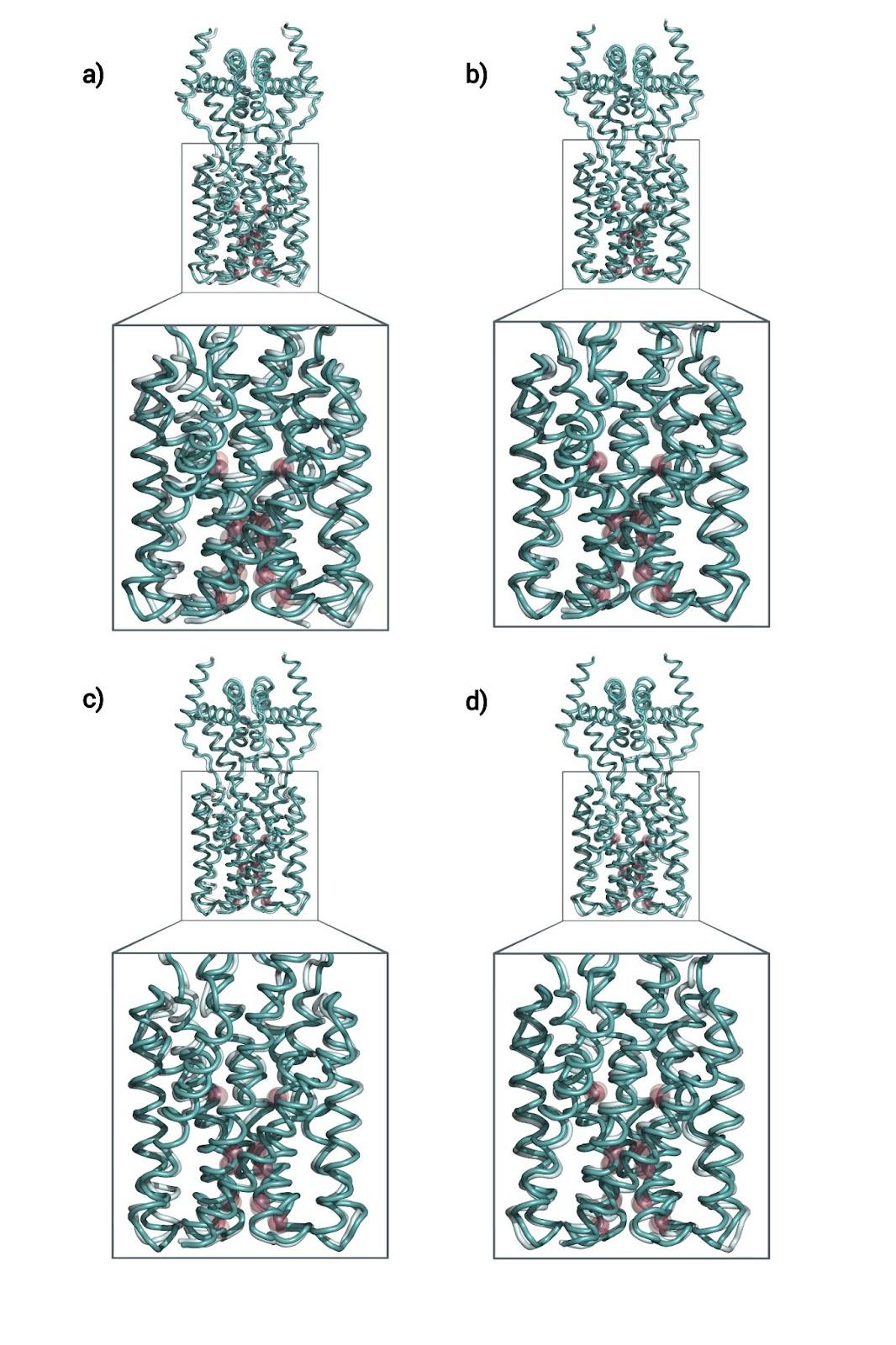


Supplemental Figure 4. Superimposition of the extremest configurations assumed assumed by OsVIT2 along the trajectory of atomic displacement generated for normal modes 35 (a), 82 (b), 87 (c) and 97 (d); spheres indicate Leu 47, 51 and 60, Asp 39 and Glu 68.


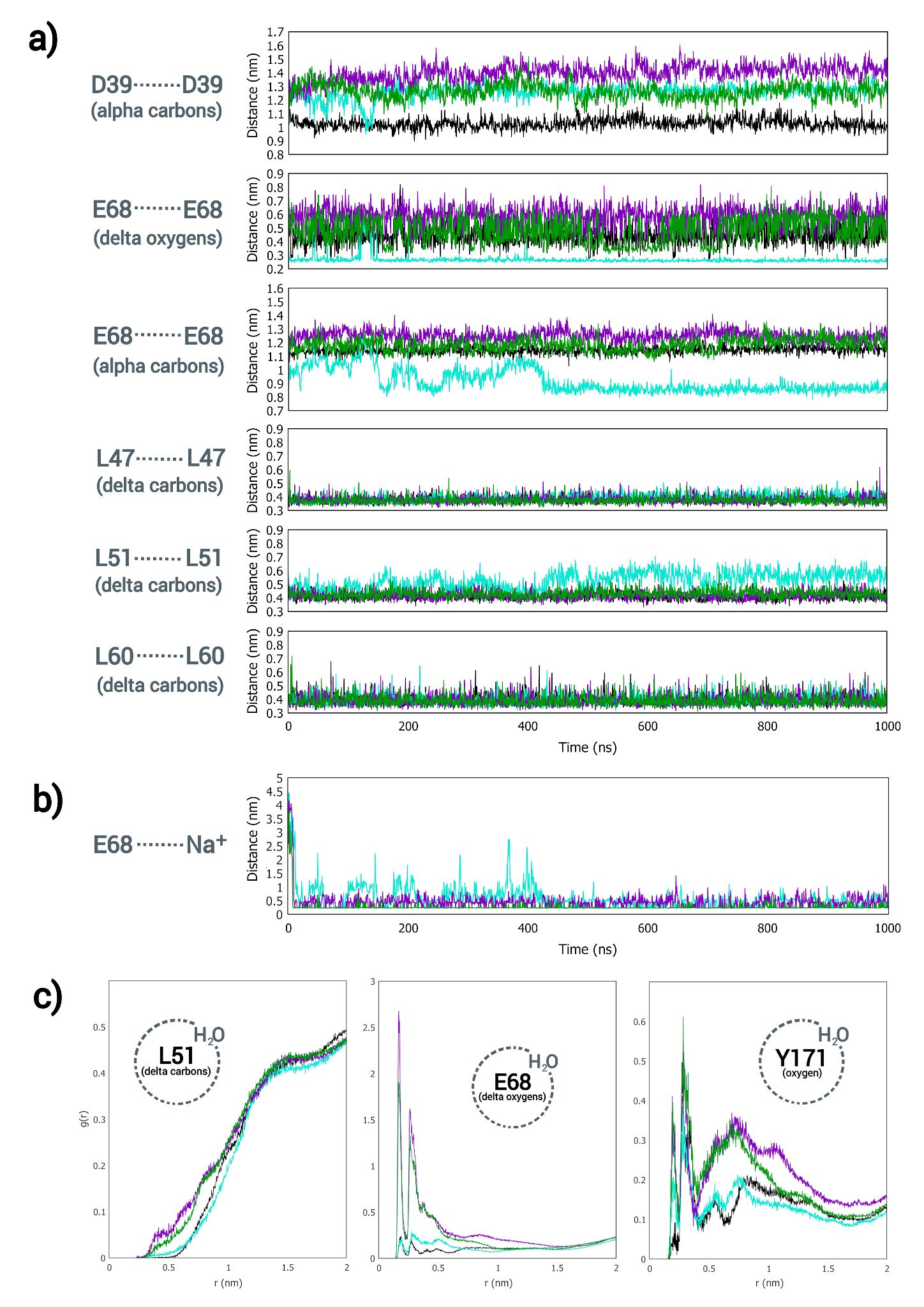


Supplemental Figure 5. a) Variation of the distance between pairs of ionizable residues that constitute the hydrophilic pocket and pairs of bulkier apolar residues that line the hydrophobic seal along replica; each line color corresponds to an protonation state of the ionizable residues that constitute the hydrophilic pocket (DE2H+ in black; DEH+ in cyan; DE in purple and DH+E in green). b) RDF plots for the same replicas, showing the hydration layers of Glu 68 and Tyr 171 but consistent lack of hydration of Leu 51. Replica 2.


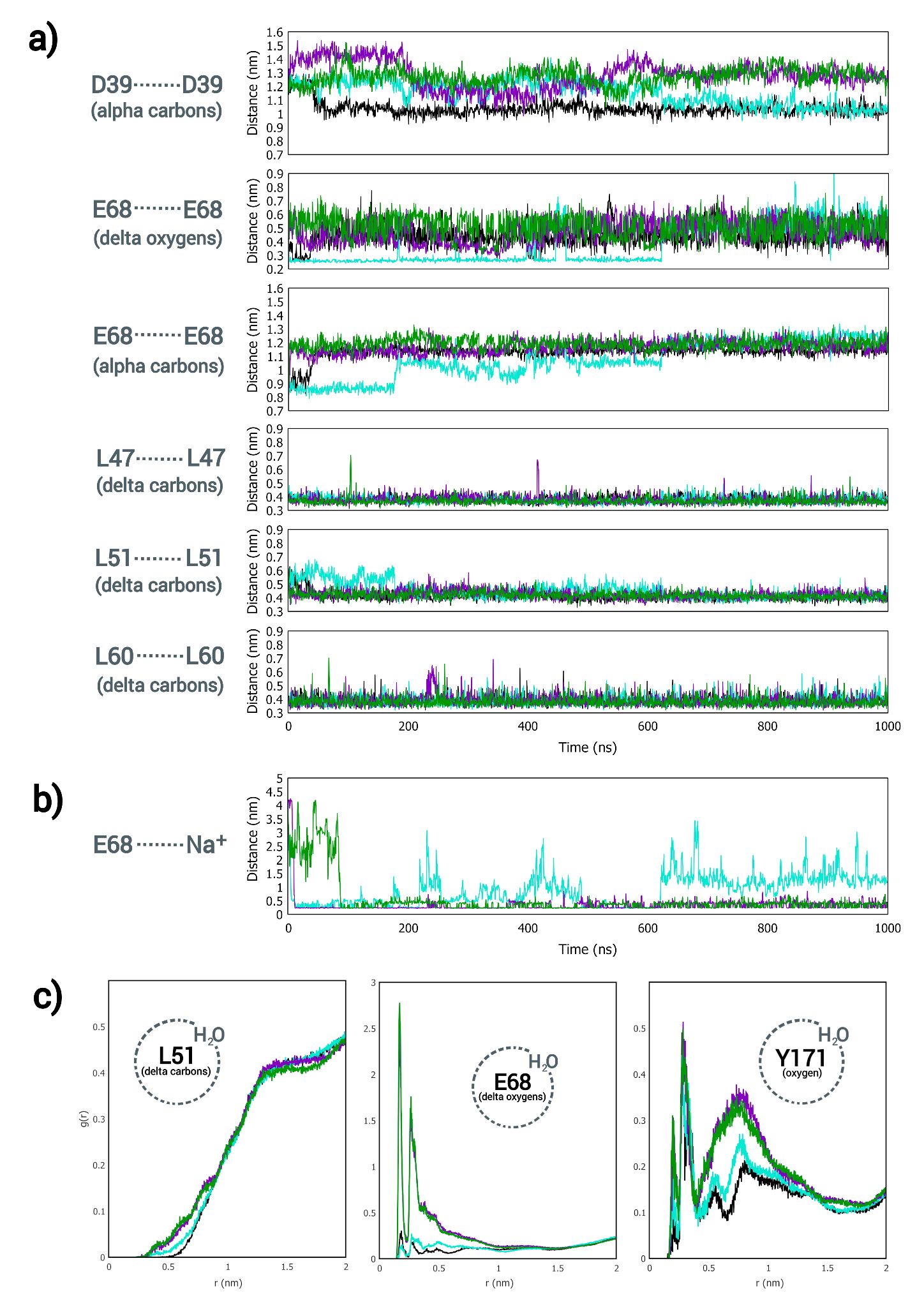


Supplemental Figure 6. a) Variation of the distance between pairs of ionizable residues that constitute the hydrophilic pocket and pairs of bulkier apolar residues that line the hydrophobic seal along replica; each line color corresponds to an protonation state of the ionizable residues that constitute the hydrophilic pocket (DE2H+ in black; DEH+ in cyan; DE in purple and DH+E in green). b) RDF plots for the same replicas, showing the hydration layers of Glu 68 and Tyr 171 but consistent lack of hydration of Leu 51. Replica 3.

|  | | | Glu 68 | | | | Asp 39 | | | | |
| --- | --- | --- | --- | --- | --- | --- | --- | --- | --- | --- | --- |
| TRAJ | FRAME | CHAIN | pKa | SC H-BOND | BB H-BOND | CI | | pKa | SD H-BOND | BB H-BOND | CI |
| DE2H+ | 200  ns | A | 9.48 | 0.0 | 0.0 | 0.91 E68 | | 6.4 | 0.0 | -0.01 D39 | 0.0 |
|  |  | B | 8.42 | -0.14 Y171 | -0.02 L47 | 0.0 | | 8.66 | 0.0 | 0.0 | 1.88 D39 |
|  | 400  ns | A | 9.43 | 0.0 | -0.01 L47 | 1.08 E68 | | 6.44 | 0.0 | 0.0 | 0.0 |
|  |  | B | 9.48 | 0.0 | 0.0 | 0.91 E68 | | 8.06 | 0.0 | 0.0 | 1.55 D39 |
|  | 600  ns | A | 9.63 | 0.0 | -0.12 L47 | 1.24 E68 | | 7.89 | 0.0 | 0.0 | 1.29 D39 |
|  |  | B | 8.26 | 0.0 | 0.0 | 0.0 | | 6.39 | 0.0 | 0.0 | 0.0 |
|  | 800  ns | A | 6.67 | -1.6 E68 | 0.0 | 0.0 | | 8.15 | 0.0 | 0.0 | 1.45 D39 |
|  |  | B | 12.29 | 1.6   E68 | 0.0 | 2.12 E68 | | 6.67 | 0.0 | 0.0 | 0.0 |
|  | 1000  ns | A | 9.38 | 0.0 | 0.0 | 1.07 E68 | | 6.29 | 0.0 | 0.0 | 0.0 |
|  |  | B | 8.28 | 0.0 | -0.04 L47 | 0.0 | | 7.84 | 0.0 | 0.0 | 1.22 D39 |
|  | min  dist  E68 | A | 12.52 | -0.37 Y171  1.6 E68 | 0.0 | 2.4 E68 | | 6.21 | 0.0 | 0.0 | 0.0 |
|  |  | B | 6.16 | -0.59 Y171  -1.6 E68 | 0.0 | 0.0 | | 6.41 | -0.21 S75 | 0.0 | 0.0 |
|  | max  dist  E68 | A | 6.96 | -0.8 Y171 | -0.01 L47 | 0.0 | | 6.56 | 0.0 | 0.0 | 0.0 |
|  |  | B | 7.16 | -0.41 Y171 | 0.0 | 0.0 | | 7.79 | -0.11 T42 | 0.0 | 1.22 E39 |
| DEH+ | 200  ns | A | 8.33 | 0.0 | 0.0 | 0.23 E68 | | 7.75 | 0.0 | 0.0 | 1.14 D39 |
|  |  | B | 6.53 | -0.8 Y171 | 0.0 | 0.0 | | 6.46 | 0.0 | 0.0 | 0.0 |
|  | 400  ns | A | 12.73 | 1.6 E68 | 0.0 | 2.4 E68 | | 6.41 | 0.0 | 0.0 | 0.0 |
|  |  | B | 6.27 | -0.15 Y171  -1.6 E68 | 0.0 | 0.0 | | 7.67 | 0.0 | 0.0 | 1.22 E39 |
|  | 600  ns | A | 8.61 | -0.59 Y171 | 0.0 | 1.45 E68 | | 6.24 | 0.0 | 0.0 | 0.0 |
|  |  | B | 6.73 | -0.8 Y171 | 0.0 | 0.0 | | 5.99 | 0.0 | 0.0 | 0.0 |
|  | 800  ns | A | 12.54 | 1.6 E68 | 0.0 | 2.4 E68 | | 6.24 | 0.0 | 0.0 | 0.0 |
|  |  | B | 6.2 | -1.6 Y171 | 0.0 | 0.0 | | 6.35 | 0.0 | 0.0 | 0.0 |
|  | 1000  ns | A | 8.88 | 0.0 | 0.0 | 1.16 E68 | | 7.01 | 0.0 | 0.0 | 0.52 D39 |
|  |  | B | 6.8 | -0.8 Y171 | 0.0 | 0.0 | | 6.28 | 0.0 | 0.0 | 0.0 |
|  | min  dist  E68 | A | 12.38 | 1.6 E68 | 0.0 | 2.4 E68 | | 6.33 | 0.0 | 0.0 | 0.0 |
|  |  | B | 5.45 | -0.8 Y171  -1.6 E68 | 0.0 | 0.0 | | 6.28 | 0.0 | 0.0 | 0.0 |
|  | max  dist  E68 | A | 7.82 | 0.0 | 0.0 | 0.0 | | 7.2 | 0.0 | 0.0 | 1.03 D39 |
|  |  | B | 6.54 | -0.8 Y171 | 0.0 | 0.0 | | 6.13 | 0.0 | 0.0 | 0.0 |

|  | | | Glu 68 | | | | | Asp 39 | | | |
| --- | --- | --- | --- | --- | --- | --- | --- | --- | --- | --- | --- |
| TRAJ | FRAME | CHAIN | pKa | SC H-BOND | BB H-BOND | CI | pKa | | SD H-BOND | BB H-BOND | CI |
| DE | 200  ns | A | 8.92 | 0.0 | 0.0 | 1.37 E68 | 6.0 | | 0.0 | 0.0 | 0.0 |
|  |  | B | 7.01 | -0.4 Y171 | 0.0 | 0.0 | 6.21 | | 0.0 | 0.0 | 0.0 |
|  | 400  ns | A | 7.19 | -0.11 Y171 | 0.0 | 0.0 | 5.93 | | 0.0 | 0.0 | 0.0 |
|  |  | B | 8.36 | 0.0 | 0.0 | 1.09 E68 | 5.68 | | 0.0 | 0.0 | 0.0 |
|  | 600  ns | A | 6.8 | -0.68 Y171 | 0.0 | 0.0 | 6.12 | | 0.0 | 0.0 | 0.0 |
|  |  | B | 9.71 | 0.0 | 0.0 | 1.61 E68 | 6.77 | | 0.0 | 0.0 | 0.4 D29 |
|  | 800  ns | A | 10.19 | 0.0 | 0.0 | 2.35 E68 | 5.94 | | 0.0 | 0.0 | 0.0 |
|  |  | B | 7.6 | 0.0 | 0.0 | 0.0 | 6.08 | | 0.0 | 0.0 | 0.0 |
|  | 1000  ns | A | 7.89 | 0.0 | 0.0 | 0.64 E68 | 6.1 | | 0.0 | 0.0 | 0.0 |
|  |  | B | 6.65 | -0.65 Y171 | 0.0 | 0.0 | 6.14 | | 0.0 | 0.0 | 0.0 |
|  | min  dist  E68 | A | 6.16 | -1.6 E68 | 0.0 | 0.0 | 6.06 | | -0.01 T42 | 0.0 | 0.0 |
|  |  | B | 11.96 | 1.6 E68 | 0.0 | 2.26 E68 | 6.06 | | 0.0 | 0.0 | 0.0 |
|  | max  dist  E68 | A | 6.42 | -0.8 Y171 | 0.0 | 0.0 | 6.1 | | 0.0 | 0.0 | 0.0 |
|  |  | B | 6.49 | -0.75 Y171 | 0.0 | 0.0 | 6.24 | | 0.0 | 0.0 | 0.0 |
| DH+E | 200  ns | A | 8.9 | 0.0 | 0.0 | 1.18 E68 | 6.28 | | 0.0 | 0.0 | 0.0 |
|  |  | B | 7.6 | 0.0 | 0.0 | 0.0 | 6.35 | | 0.0 | 0.0 | 0.0 |
|  | 400  ns | A | 6.64 | -0.8 Y171 | 0.0 | 0.0 | 6.14 | | 0.0 | 0.0 | 0.0 |
|  |  | B | 7.32 | 0.0 | 0.0 | 0.5 E68 | 6.38 | | 0.0 | 0.0 | 0.0 |
|  | 600  ns | A | 8.98 | 0.0 | 0.0 | 1.48 E68 | 6.49 | | 0.0 | 0.0 | 0.0 |
|  |  | B | 7.06 | -0.45 Y171 | 0.0 | 0.0 | 6.22 | | 0.0 | 0.0 | 0.0 |
|  | 800  ns | A | 6.99 | -0.6 Y171 | 0.0 | 0.0 | 6.58 | | 0.0 | 0.0 | 0.24 D39 |
|  |  | B | 10.33 | 0.0 | 0.0 | 2.07 E68 | 6.17 | | 0.0 | 0.0 | 0.0 |
|  | 1000  ns | A | 7.96 | 0.0 | 0.0 | 0.54 E68 | 5.91 | | 0.0 | 0.0 | 0.0 |
|  |  | B | 6.6 | -0.8 Y171 | 0.0 | 0.0 | 5.89 | | 0.0 | 0.0 | 0.0 |
|  | min  dist  E68 | A | 6.24 | -1.6 E68 | 0.0 | 0.0 | 6.32 | | 0.0 | -0.03 D39 | 0.0 |
|  |  | B | 11.45 | 1.6 E68 | 0.0 | 1.88 E68 | 6.41 | | 0.0 | -0.01 E39 | 0.0 |
|  | max  dist  E68 | A | 6.73 | -0.43 Y171 | 0.0 | 0.0 | 5.73 | | 0.0 | 0.0 | 0.0 |
|  |  | B | 6.46 | -0.8 Y171 | 0.0 | 0.0 | 6.09 | | 0.0 | 0.0 | 0.0 |

Supplemental Figure 7. PKa values, side chain (SC) and backbone (BB) hydrogen bonds and coulombic interactions (CI) established by Glu 68 and Asp 39 for configurations extracted from four trajectories with different protonation states; the frames were taken every 200 ns or were selected for corresponding to the minimal and maximal distance between the Glu 68 pair.


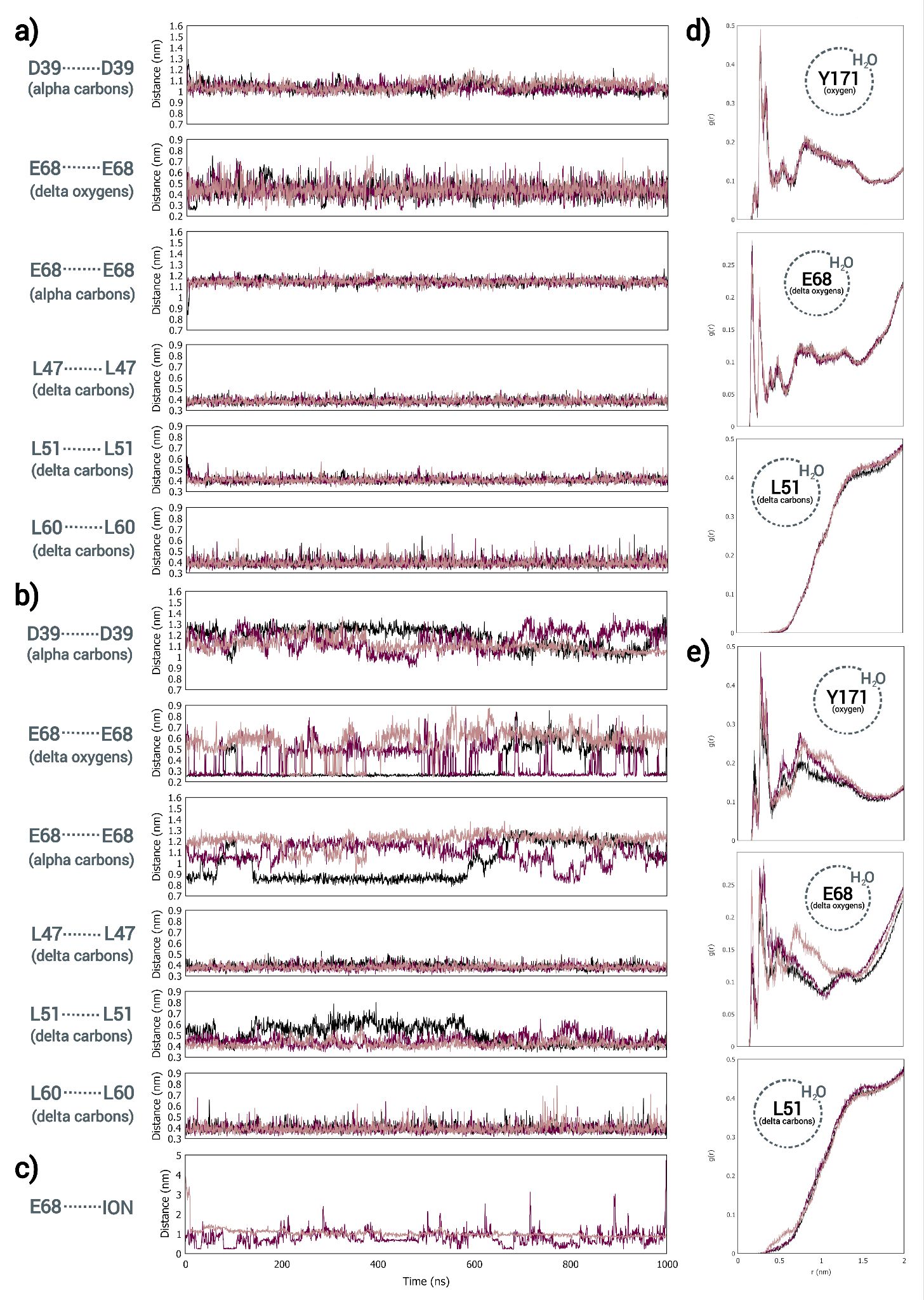


Supplemental Figure 8. Variation of the distance between pairs of ionizable residues that constitute the hydrophilic pocket and pairs of bulkier apolar residues that line the hydrophobic seal along 1 μs long MD simulations for DE2H+ in black, DE2H+6F in dark pink and DE2H+28F in light pink(a) and DEH+ in black, DEH+6F in dark pink and DEH+28F in light pink (b); each color corresponds to an occupation state of the MBS. c) Variation of the distance between Glu 68 and the nearest Na+ (in the case of DEH+6F in dark pink) or Fe2+ (in the case of DEH+28F in light pink). The RDF plots for DE2H+ (black), DE2H+6F (dark pink) and DE2H+28F (light pink) (d) and DEH+ (black), DEH+6F (dark pink) and DEH+28F (light pink) (e) are also shown.


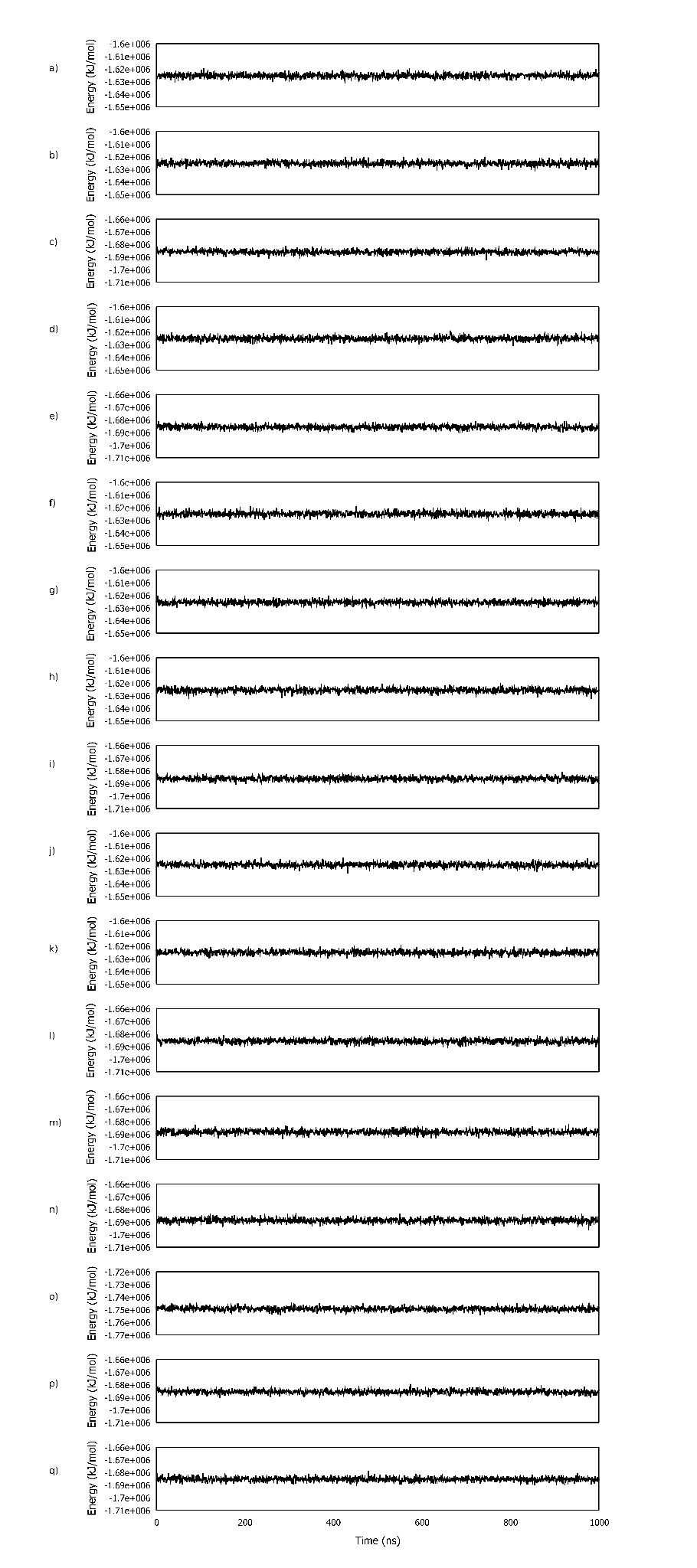


Supplemental Figure 9. Variation of the potential energy of the systems along 1 μs long MD simulations for DE2H+ replicas (a, b and c), DEH+ replicas (d, e and f), DE replicas (g, h and i), DH+E replicas (j, k and l), DE2H+28F (m), DEH+28F (n), DE28F (o), AE2H+ (p) and AEH+ (q).
